# Identifying RNA N6-Methyladenine Sites in Three Species Based on a Markov Model

**DOI:** 10.3389/fgene.2021.650803

**Published:** 2021-03-19

**Authors:** Cong Pian, Zhixin Yang, Yuqian Yang, Liangyun Zhang, Yuanyuan Chen

**Affiliations:** College of Science, Nanjing Agricultural University, Nanjing, China

**Keywords:** RNA N6-methyladenine sites, second-order Markov model, codons biases, transfer probability matrix, web tool

## Abstract

N6-methyladenosine (m6A), the most common posttranscriptional modification in eukaryotic mRNAs, plays an important role in mRNA splicing, editing, stability, degradation, etc. Since the methylation state is dynamic, methylation sequencing needs to be carried out over different time periods, which brings some difficulties to identify the RNA methyladenine sites. Thus, it is necessary to develop a fast and accurate method to identify the RNA N6-methyladenosine sites in the transcriptome. In this study, we use first-order and second-order Markov models to identify RNA N6-methyladenine sites in three species (*Saccharomyces cerevisiae*, mouse, and *Homo sapiens*). These two methods can fully consider the correlation between adjacent nucleotides. The results show that the performance of our method is better than that of other existing methods. Furthermore, the codons encoded by three nucleotides have biases in mRNA, and a second-order Markov model can capture this kind of information exactly. This may be the main reason why the performance of the second-order Markov model is better than that of the first-order Markov model in the m6A prediction problem. In addition, we provide a corresponding web tool called MM-m6APred.

## Introduction

To date, more than 160 types of RNA modifications have been discovered (Zhao et al., 2019). In these modifications, N6-methyladenosine (m6A) is the most common and abundant one existing in various species. It is closely associated with diverse biological processes, such as RNA localization and degradation (Wang et al., 2014), RNA structural dynamics (Roost et al., 2015), alternative splicing (Liu et al., 2015), and primary microRNA processing (Alarcón et al., 2015). Thus, identification of m6A sites is of great importance for better understanding their function and mechanisms (Chen et al., 2015). In the past few years, high-throughput experimental methods, such as MERIPP (Geula et al., 2015) and M6ASeq (Meyer et al., 2012), have been used to identify m6A modifications, but these methods have some limitations: (1) The location of the m6A site cannot be accurately located; (2) the cost is high; and (3) they are not applicable for the large-scale identification of m6A sites. Hence, it is highly desirable to develop a fast and accurate computational method for the identification of m6A sites (Dominissini et al., 2012).

Currently, there are several effective methods for predicting m6A sites based on machine learning, mainly including iRNA-Methyl (Chen et al., 2015), SRAMP (Zhou et al., 2016), M6AMRFS (Qiang et al., 2018), M6APred–EL (Wei et al., 2018), pm6A-CNN (Roost et al., 2015), and iN6-Methyl (Nazari et al., 2019) etc. The above methods actually use the physical and chemical properties of nucleotides in various species, such as the nucleotide frequency at specific locations and the chemical properties of nucleotides, to extract features and predict m6A sites. However, none of these methods can capture the correlation between adjacent nucleotides well, while the Markov model can model this kind of correlation. In fact, Pian et al. (2020) used a first-order Markov model to predict the DNA N6-methyladenine sites. Recently, we proposed a method to predict DNA 4mC sites based on the second-order Markov model (Yang et al., 2020). Later, we found that the second-order Markov model is more suitable for predicting the methylation sites of RNA m6A because of the biases of the triplet codons in mRNA. The main purpose of this article is to provide a more accurate prediction tool of m6A.

Based on this idea, we used a second-order Markov model to identify the m6A sites of RNA. The m6A data of the three species of *Saccharomyces* ce*revisiae*, mouse, and *Homo sapiens*, were used to evaluate our model. The results show that the prediction performances of the first-order Markov model and the second-order Markov model are significantly better than those of the other four existing prediction tools. In addition, the second-order Markov model outperforms the first-order Markov model, which indicates that the second-order Markov model can capture the codon bias in mRNA well. This suggests that second-order Markov may be able to characterize the codon bias in mRNA.

## Materials and Methods

### Benchmark Datasets

In this study, we used three benchmark datasets from three different species: *S. cerevisiae* (Chen et al., 2015), mouse (Dominissini et al., 2012), and *H. sapiens* (Chen et al., 2017). The corresponding number of positive samples was 1,300, 725, and 1,130. There were as many negative samples as positive samples. Table 1 shows the details of these data. For the three benchmark datasets, the positives were the sequences centered with true m6A sites, while the negatives were the sequences centered with adenines but without any m6A peaks detected. The datasets can be downloaded from the following website^1^.

**TABLE 1:** Details of benchmark datasets.

Type	Positive	Negative	Total	Length
Yeast cells	1,300	1,300	2,600	51 nt
Mouse	725	725	1,450	41 nt
Homo sapiens	1,130	1,130	2,260	41 nt

### Model Construction

A Markov model is a stochastic process where the next variable depends on only the most recent variable(s) instead of all the previous variables. In this sequence information study, we first model a sequence as a first-order Markov chain, and the current nucleotide depends on the previous nucleotide only. More specifically, for the m6A sequences of positive samples in the training data, we first calculate the initial probability $P_{S_{1}}^{P}(P_{A}^{P}$, $P_{G}^{P}$, $P_{C}^{P}$, $P_{U}^{P})$ of the initial state S1 nucleotide being A, G, C or U, respectively. Then, we need to calculate the transfer probability $P_{S_{n}-S_{n+1}}^{P_{n}}$of the current nucleotide state to the next state individually from the initial state S_1_ (for example, $\begin{array}{c} P_{G-A}^{P_{2}} \end{array}$ represents the probability that nucleotide G in state S_2_ transfers to nucleotide A in state S_3_).

Thus, we can obtain the probability of the occurrence of the four nucleotides in the initial state and the transfer probability matrix of each state except the last one. Similarly, for the negative sequences of non-m6A, the probability of the occurrence of the corresponding four nucleotides in the initial state and the transition probability matrix can also be obtained. Therefore, two Markov models are trained based on the m6A sequences and non-m6A sequences in the training dataset.

In the process of prediction, we need to select the probability values according to the nucleotide arrangement of the sequence, including the initial state probability and the corresponding transfer probability from the positive and negative Markov models in the previous step. Then, we calculate the products of positive and negative probability values. Finally, we calculate the ratio of the positive product and negative product. If the ratio is greater than 1, the sequence is considered a m6A sample. Otherwise, it is considered a non-m6A sample.

**FIGURE 1:**
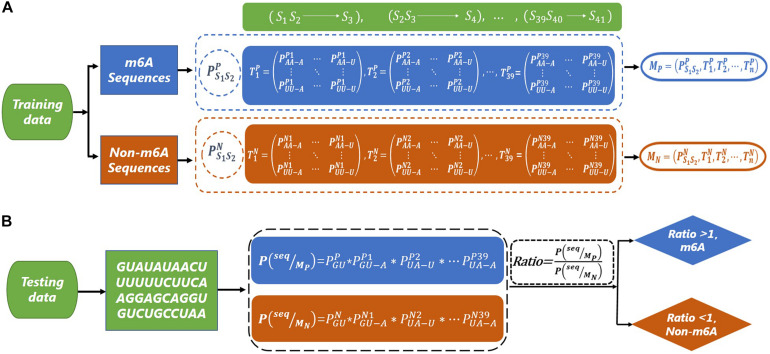
The flow chart of m6A site prediction. **(A)** The construction of second-order Markov model (*M*_*P*_ and *M*_*N*_) based on m6A sequence and non-m6A sequence. **(B)** The prediction for a test sequence. The sequence “GUAUAUAACUUUUUUCUUCAAGGAGCAGGUGUCUGCCUAA” is used as an example to explain the prediction process.

Since there is a bias in the codon of mRNA (Kurland, 1991; Quax, 2015), we consider using a second-order Markov model to capture this bias. The flowcharts of the training and testing of the second-order Markov model are shown in Figure 1. For the m6A sequences, we first calculate the initial probability $P_{S_{1}\,S_{2}}^{P}\,\left(P_{A\,A}^{P},P_{A\,G}^{P},\ldots ,P_{U\,U}^{P}\right)$ of the first dinucleotide. Then, we need to calculate the transfer probability$P_{S_{n}\,S_{n+1}-S_{n+2}}^{P_{n}}$of the current dinucleotide (*S_*n*_ S_*n*_*_+_*_1_*) to the next nucleotide (*S*_*n*+_*_2_*) (for example, $P_{A\,A-A}^{P\,1}$represents the probability of state S_1_S_2_ transferring to S_3_, where the nucleotide of state S_1_S_2_ is AA, and the nucleotide of state S_3_ is A). Thus, 39 transfer probability matrices with 16 rows and four columns can be obtained. Similarly, the initial probability and transfer probability can be obtained for non-m6A sequences. Therefore, two Markov models (*M_P* and *M_N*) are similarly trained based on the m6A sequences and non-m6A sequences in the training dataset. Taking the sequence “seq = GUAUAUAACUUUUUUCUUCAAGGAGCAGGUGUC UGCCUAA” as an example, the probabilities *P*(*seq|*M_P**) and *P*(*seq*|*M_N*) of the sequence “seq” under models *M_P* and *M_N* are obtained, respectively. Then, the value of *Ratio* = *P(seq|M_*P*_)/P(seq|M_*N*_)* can be used to determine the class of “seq,” where

$$P\,\left(s\,e\,q|M_{P}\right)=P_{G\,U}^{P}\times P_{G\,U-A}^{P_{1}}\times P_{U\,A-U}^{P_{2}}\times \ldots \times P_{U\,A-A}^{P_{39}},$$

and

$$P\,\left(s\,e\,q|M_{N}\right)=P_{G\,U}^{N}\times P_{G\,U-A}^{N_{1}}\times P_{U\,A-U}^{N_{2}}\times \ldots \times P_{U\,A-A}^{N_{39}},$$

If the *Ratio* > 1, “seq” is classified as a m6A sequence; otherwise, it is classified as a non-m6A sequence.

### Performance Evaluation

Ten-fold cross-validation was used to assess the reliability of the method. In the performance evaluation, the sensitivity (Sn), specificity (Sp), accuracy (ACC), and Mathew’s correlation coefficient (MCC) were calculated. They are formulated as follows:

$$S_{n}=\frac{T_{P}}{T_{P}+F_{N}},$$

$$S_{p}=\frac{T_{N}}{T_{N}+F_{P}},$$

$$A\,C\,C=\frac{T_{P}+T_{N}}{T_{P}+T_{N}+F_{P}+F_{N}},$$

$$M\,C\,C=\frac{T_{P}+T_{N}-F_{P}\times F_{N}}{\sqrt{\left(T_{P}+F_{P}\right)\times \left(T_{N}+F_{N}\right)\times \left(T_{P}+F_{N}\right)\times \left(T_{N}+F_{P}\right)}}$$

where T_*P*_, T_*N*_, F_*P*_, and F_*N*_ denote true positive, true negative, false positive, and false negative, respectively. *S*_*n*_ measures the predictive ability of a predictor for positive samples, while *S*_*p*_ measures the predictive ability of a predictor for negative samples. *ACC* and *MCC* are two metrics measuring the overall performance of a predictor.

## Results and Discussion

### Representation and Illustration of ($P_{S_{n}\,S_{n+1}-S_{n+2}}^{P_{n}}/P_{S_{n}\,S_{n+1}-S_{n+2}}^{N_{n}}$)

**FIGURE 2:**
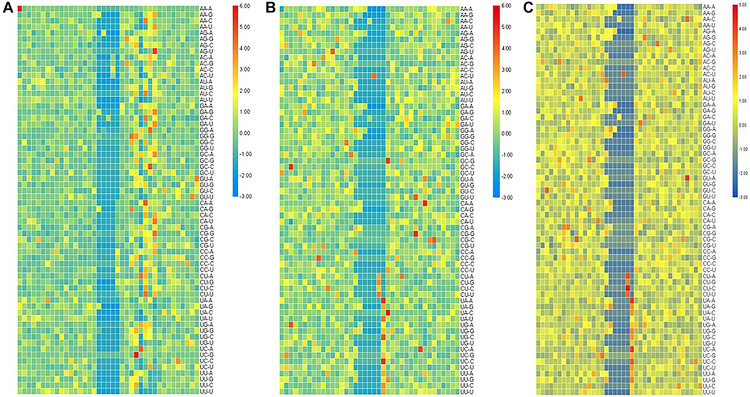
The heat map of standardized quotient of the transfer probabilities of the three types of species. The heat map of standardized quotient of the transfer probabilities of the three types of species. **(A)** *Saccharomyces cerevisiae* cells, **(B)** mouse, and **(C)** *Homo sapiens.*

For the second-order Markov model, the heat map of the quotient matrix ($P_{S_{n}\,S_{n+1}-S_{n+2}}^{P_{n}}/P_{S_{n}\,S_{n+1}-S_{n+2}}^{N_{n}}$) of second-order transfer probability of m6A samples divided by the second-order transfer probability of non-m6A samples is shown in Figure 2. In order to facilitate comparison, the results of heat map were standardized. The results show that there is a significant difference in the transfer probability of nucleotides at some positions between the positive and negative samples. This indicated that the second-order Markov chain is informative for predicting sequences containing m6A sites.

We also plotted the line charts of transfer probability of the second-order Markov model (Shown in Supplementary Material 1). Similar to the first-order Markov model, the transfer probability of positive samples is significantly different from that of negative samples in the second-order Markov model. Furthermore, the number of significant different sits in the second-order Markov model are obviously greater than that in the first-order Markov model from the line charts in Supplementary Material 2. It indicates that more information is provided in the second-order Markov model to help determine the type of sequences.

### The Distribution of Ratios in the Positive and Negative Sample Sets

**FIGURE 3:**
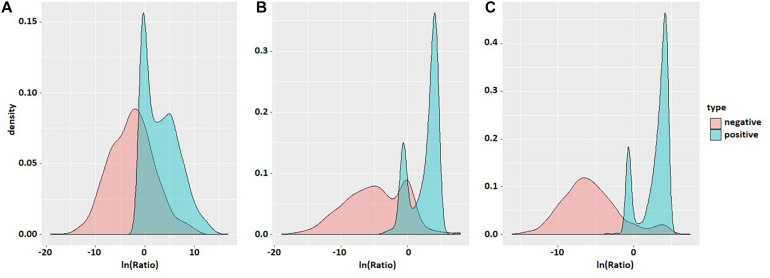
Probability density maps of *ln(Ratio)* values of the three species. The three density maps **(A)**, **(B)**, and **(C)** correspond to *S. cerevisiae*, mouse, and *H. sapiens*. Red is negative and blue is positive.

Probability density maps of *ln(Ratio)* values for three species based on the second-order transfer probability products are shown in Figure 3. It can be found that in each species, the distribution of *ln(Ratio)* is very different between positive and negative samples, except for a small amount of overlap in the probability density graphs. The *Ratio* value of positive samples is significantly greater than that of negative samples, which enables the positive and negative samples to be divided accurately.

### Comparison and Analysis

**FIGURE 4:**
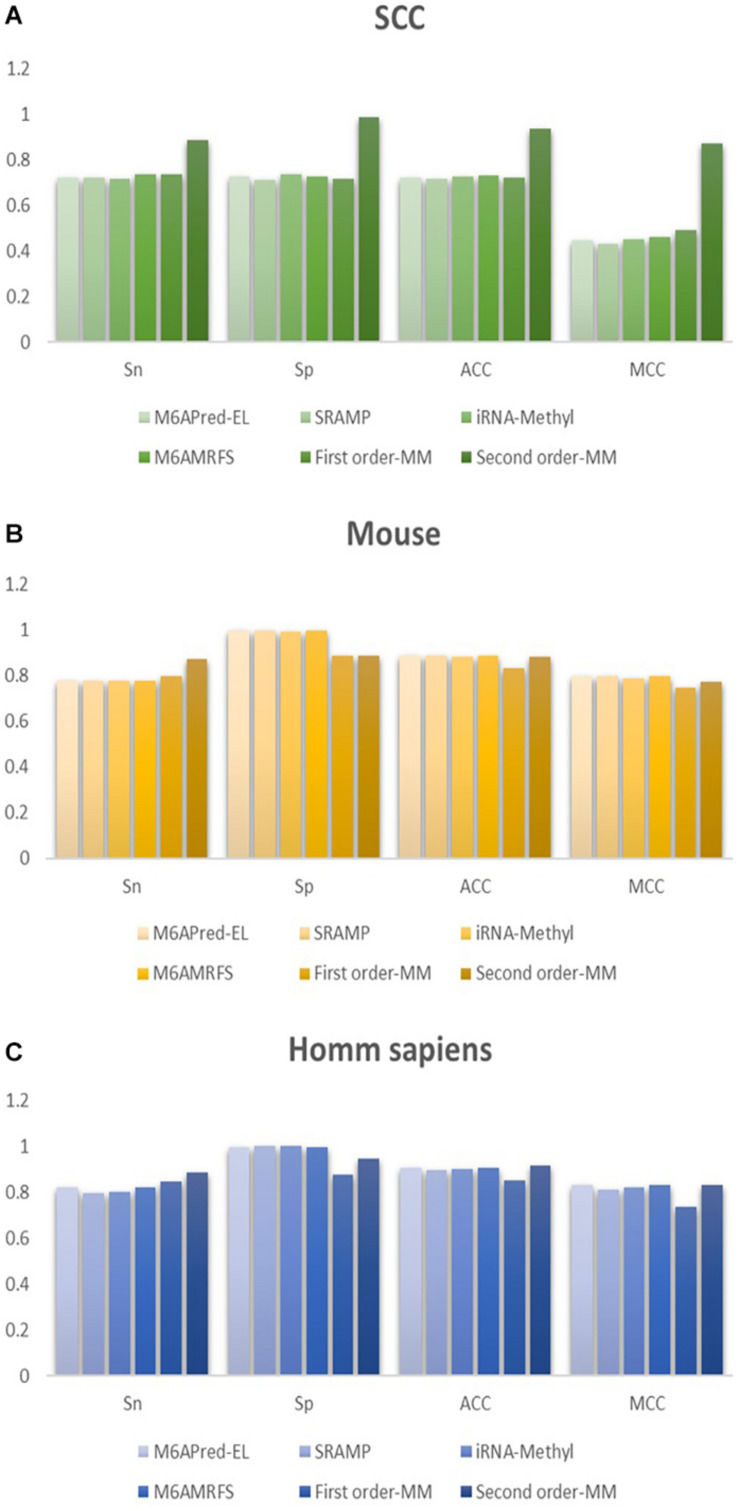
Comparison of the prediction effect of the six methods in **(A)** *S. cerevisiae*, **(B)** mouse, and **(C)** *H. sapiens.*

To evaluate our Markov model, we compared the performance of the two methods based on the Markov model with those of other m6A classifiers, including iRNA-Methyl, SRAMP, M6AMRFS, and M6APred-EL. Table 2 and Figure 4 show the prediction results of various methods (10-fold cross validation was used in all methods).

**TABLE 2:** Evaluation data comparison table of six methods in **(A)** *S. cerevisiae*, **(B)** mouse, and **(C)** *H. sapiens*.

A	*S*_*n*_ (%)	*S*_*p*_ (%)	ACC (%)	MCC
M6APred-EL	72	72.69	72.34	44.68
SRAMP	71.92	71.38	71.65	43.31
iRNA-Methyl	71.69	73.45	72.57	45.15
M6AMRFS	73.45	72.84	73.14	46.29
First order-MM	73.85	71.69	72.30	49.23
Second order-MM	88.46	98.46	93.46	87.36

**B**	***S*_*n*_ (%)**	***S*_*p*_ (%)**	**ACC (%)**	**MCC**

M6APred-EL	77.79	1	88.90	79.79
SRAMP	77.79	1	88.90	79.79
iRNA-Methyl	77.66	99.31	88.48	78.84
M6AMRFS	77.79	1	88.90	79.79
First order-MM	79.98	88.88	83.55	74.85
Second order-MM	87.50	88.88	88.29	77.45

**C**	***S*_*n*_ (%)**	***S*_*p*_ (%)**	**ACC (%)**	**MCC**

M6APred-EL	82.04	99.73	90.89	83.08
SRAMP	79.65	1	89.82	81.35
iRNA-Methyl	80.35	1	90.18	81.95
M6AMRFS	81.95	99.82	90.89	83.11
First order-MM	84.60	87.50	85.00	73.85
Second order-MM	86.46	94.69	90.58	81.43

**FIGURE 5:**
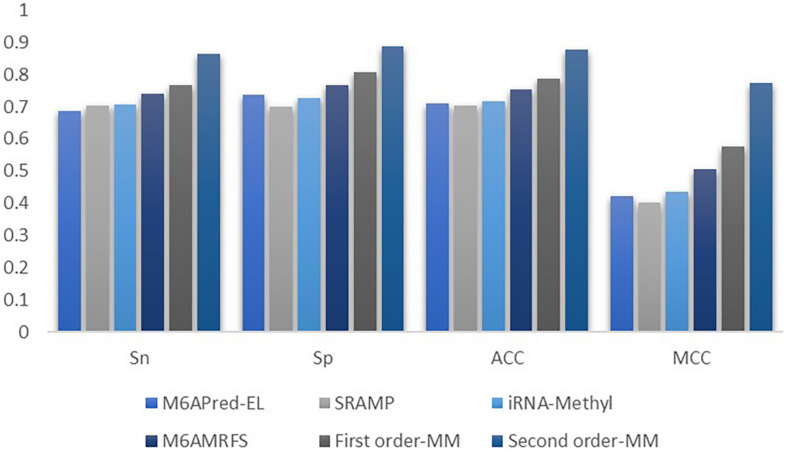
Comparison of the prediction effect of m6A in mice based on the m6Avar database.

It can be found that the two methods based on the Markov model in m6A types of sequence identification had better or equal classification effects than several kinds of classifiers and that the second-order Markov model performed much better than the first-order Markov model in each aspect. It is noteworthy that Sp in several other methods is 100% in the species of mouse and *H. sapiens*, while the Sp of our method is close to 90% on average. Therefore, we checked these non-m6A data and found that the selection of negative sample data in the original literature ^[12]^ is unreasonable. The states S_22_ of the negative samples in mouse and human are all C, and the states S_20_ are all A or G. This is the reason why Sp of other methods can reach 100%. To evaluate our method more fairly, we downloaded 725 m6A sequences of mice from the m6Avar database, and the same number of sequences were randomly selected from the non-m6A sequences of the dbSNP database as negative samples. We used these data to retrain new models and carried out 10-fold cross validation in all methods. The performance results of all the above methods are shown in Table 3 and Figure 5. The results indicate that all the performance metrics based on the two Markov model are high. And the second-order Markov model still performed much better than the first-order Markov mode.

**TABLE 3:** Comparison of the prediction effect of m6A in mice based on the m6Avar database.

Method	*S*_*n*_ (%)	*S*_*p*_ (%)	*ACC* (%)
M6APred-EL	76.42	77.35	75.49
SRAMP	72.03	72.29	71.77
iRNA-Methyl	73.45	74.72	72.18
M6AMRFS	76.58	76.89	76.27
First order-MM	78.15	80.01	80.14
Second order-MM	**86.22**	**87.13**	**85.32**

*The best performance in the respective part appears bold in the table.*

### Web-Server Implementation

To facilitate the use of the Markov model to identify RNA m6A sites, the user-friendly web server MM-m6APred has been provided. It is freely available at^2^. Our tool can handle RNA sequences of 41 bp or longer. Users can either paste RNA sequences into the text area or upload a FASTA format file.

## Conclusion

Accurate identification of the m6A site is a necessary step in the study of its biological function. In this study, we used first-order and second-order Markov models to predict the m6A sites of three species. The results show that our method is better than the other four existing prediction tools. This shows that the Markov model can capture the correlation between neighboring nucleotides well. Considering the biases of the codons in mRNA, the second-order Markov model is used to capture these biases. The results show that the prediction performance of the second-order Markov model is significantly better than that of the first-order Markov model. In addition, we also provide the online prediction web tool of m6A, with code available to download (see text footnote 2).

## Data Availability Statement

Publicly available datasets were analyzed in this study. This data can be found here: http://server.malab.cn/M6AMRFS.

## Author Contributions

CP and ZY contributed equally to this work. All authors read and approved the final manuscript.

## Conflict of Interest

The authors declare that the research was conducted in the absence of any commercial or financial relationships that could be construed as a potential conflict of interest.

## Footnotes

1 http://server.malab.cn/M6AMRFS/

2 http://www.pianlab.cn/m6APred/
