# Supplementary material for: Identifying RNA N6-Methyladenine Sites in Three Species Based on a Markov Model
Source: Front Genet. 2021 Mar 19;12:650803. doi: 10.3389/fgene.2021.650803 (PMC8017269; doi:10.3389/fgene.2021.650803)
Supplement: Supplementary file 1 [file Presentation_1.zip › Supplementary Figures S1 - S4.PDF]

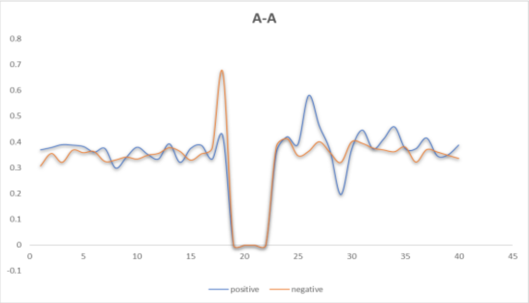
A-A
positive
negative

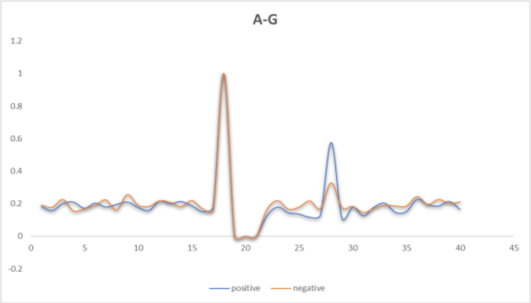
A-G
positive
negative

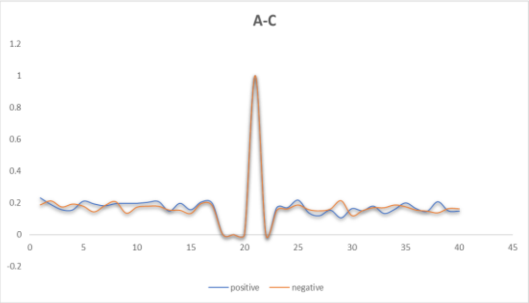
A-C
positive
negative

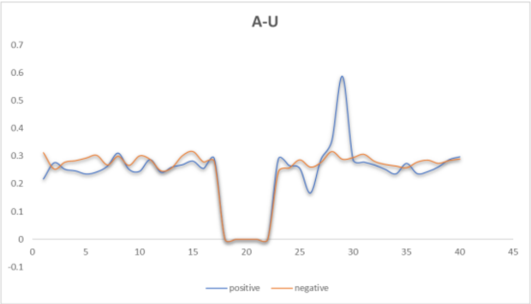
A-U
positive
negative

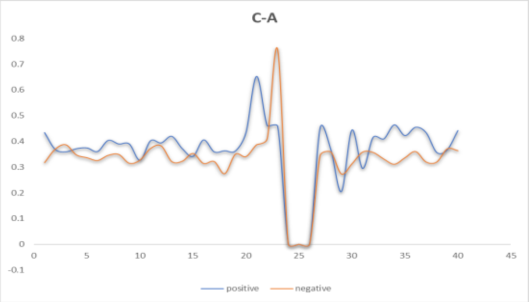
C-A
positive
negative

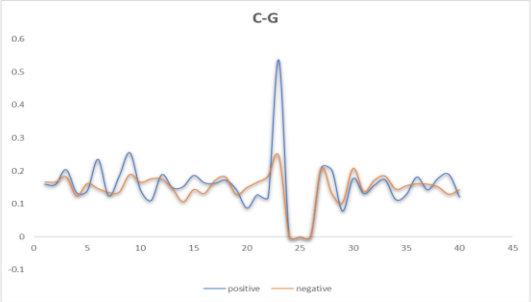
C-G
positive
negative

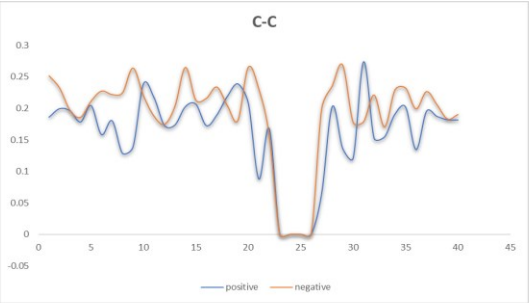
C-C
positive
negative

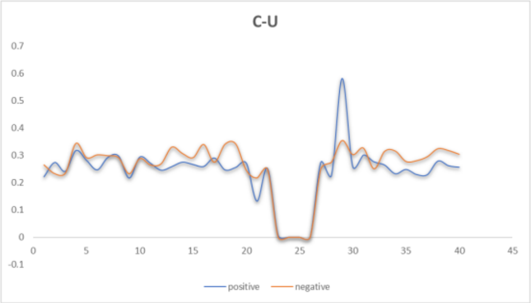
C-U
positive
negative

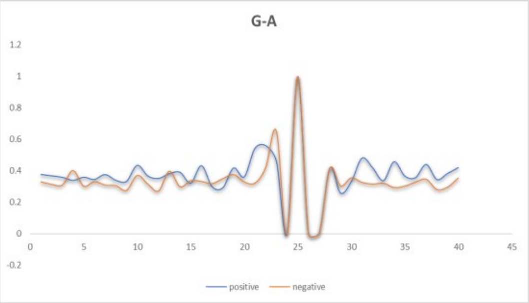
G-A
positive
negative

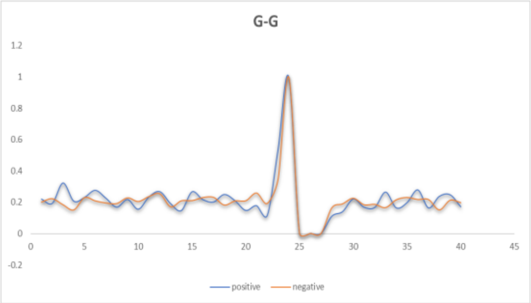
G-G
positive
negative

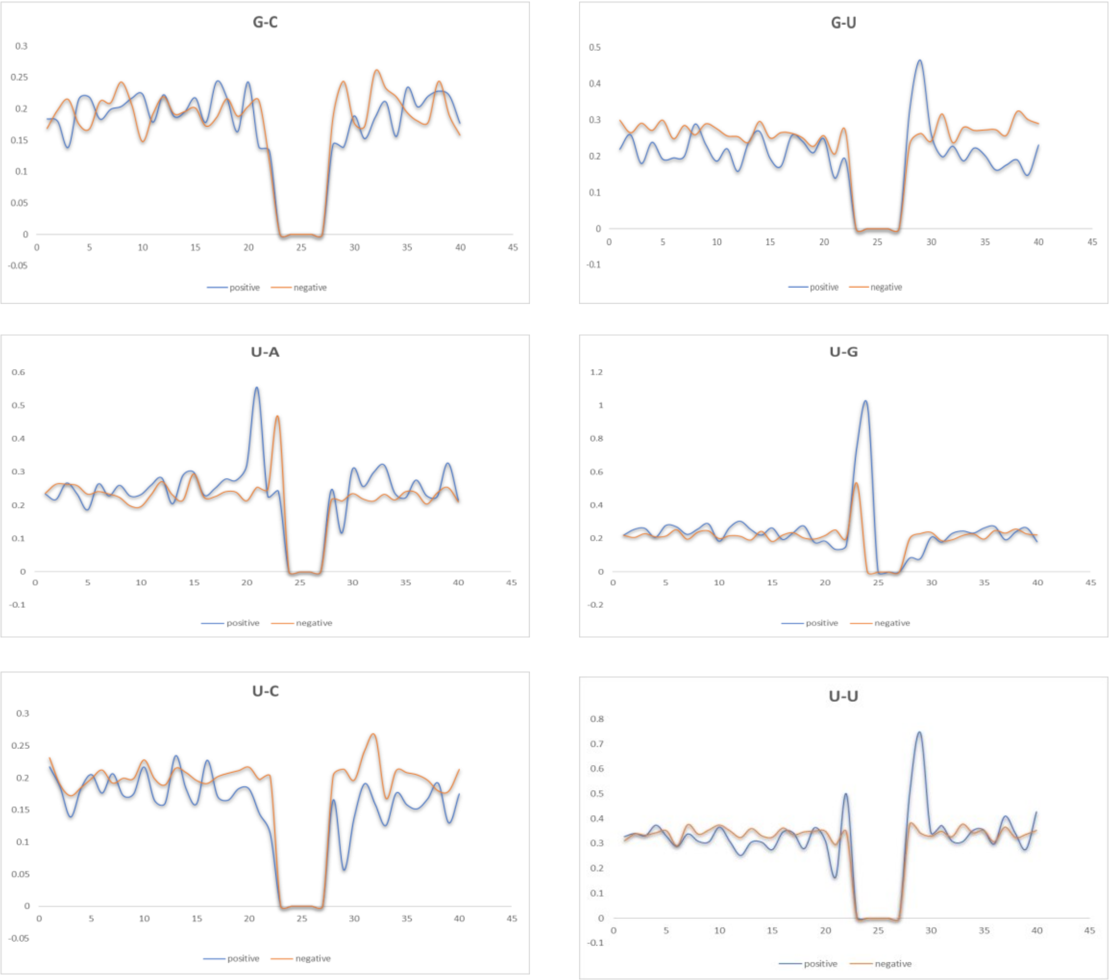

Figure S1 First-order transition probabilities of Saccharomyces data between adjacent nucleotides at all sites. The blue and red lines represent the m6A and non-m6A sequences, respectively.

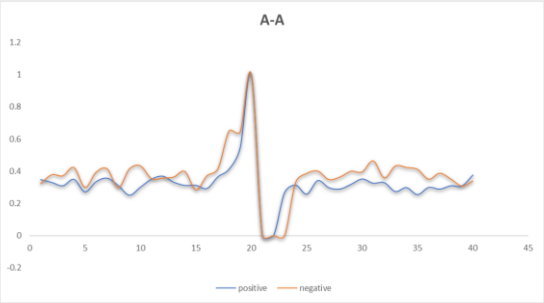
A-A
1.2
1
0.8
0.6
0.4
0.2
0
-0.2
0 5 10 15 20 25 30 35 40 45
positive
negative

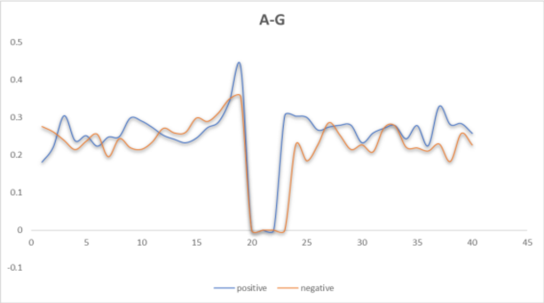
A-G
0.5
0.4
0.3
0.2
0.1
0
-0.1
0 5 10 15 20 25 30 35 40 45
positive
negative

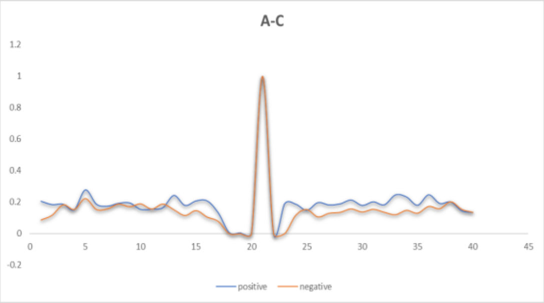
A-C
1.2
1
0.8
0.6
0.4
0.2
0
-0.2
0 5 10 15 20 25 30 35 40 45
positive
negative

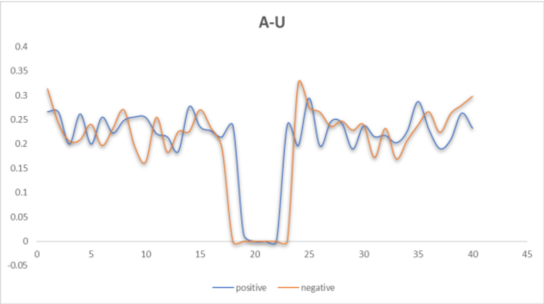
A-U
0.4
0.35
0.3
0.25
0.2
0.15
0.1
0.05
0
-0.05
0 5 10 15 20 25 30 35 40 45
positive
negative

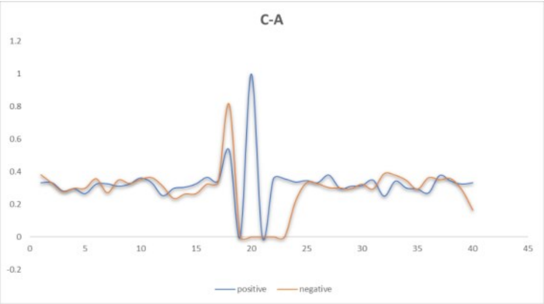
C-A
1.2
1
0.8
0.6
0.4
0.2
0
-0.2
0 5 10 15 20 25 30 35 40 45
positive
negative

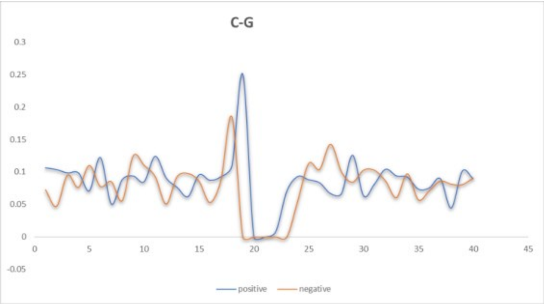
C-G
0.3
0.25
0.2
0.15
0.1
0.05
0
-0.05
0 5 10 15 20 25 30 35 40 45
positive
negative

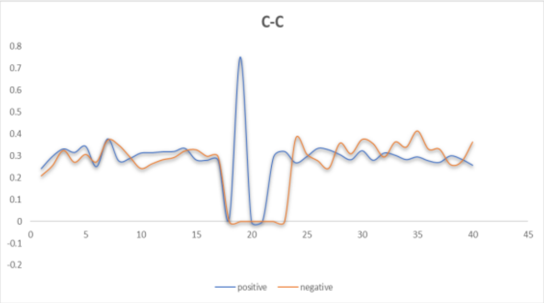
C-C
0.8
0.7
0.6
0.5
0.4
0.3
0.2
0.1
0
-0.1
-0.2
0 5 10 15 20 25 30 35 40 45
positive
negative

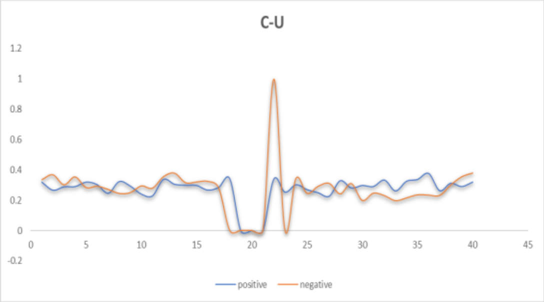
C-U
1.2
1
0.8
0.6
0.4
0.2
0
-0.2
0 5 10 15 20 25 30 35 40 45
positive
negative

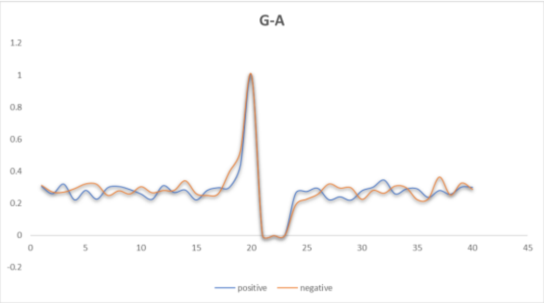
G-A
1.2
1
0.8
0.6
0.4
0.2
0
-0.2
0 5 10 15 20 25 30 35 40 45
positive
negative

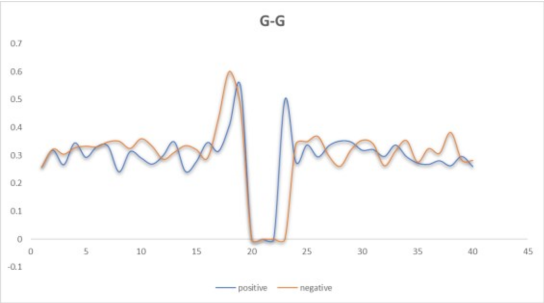
G-G
0.7
0.6
0.5
0.4
0.3
0.2
0.1
0
-0.1
0 5 10 15 20 25 30 35 40 45
positive
negative

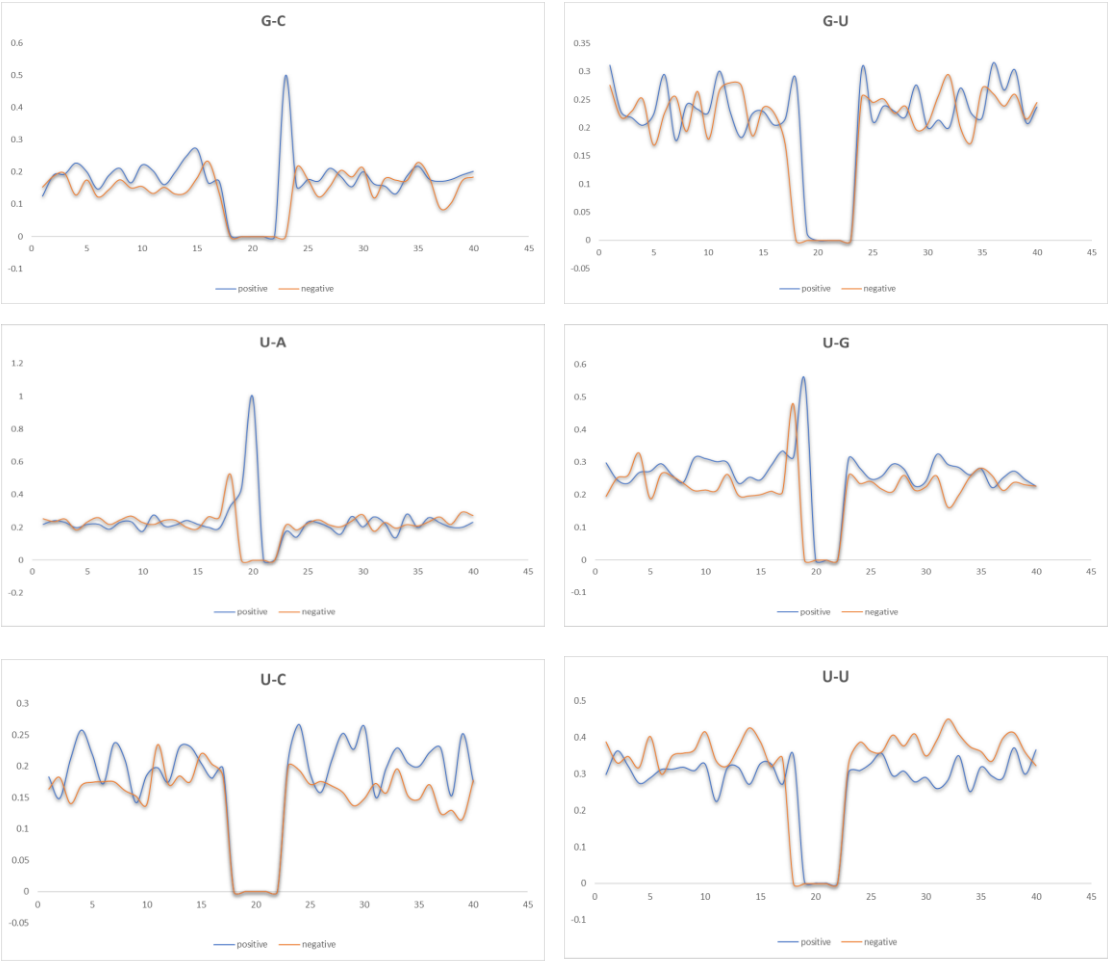

Figure S2 First-order transition probabilities of Mouse data between adjacent nucleotides at all sites. The blue and bred lines represent the m6A and non-m6A sequences, respectively.

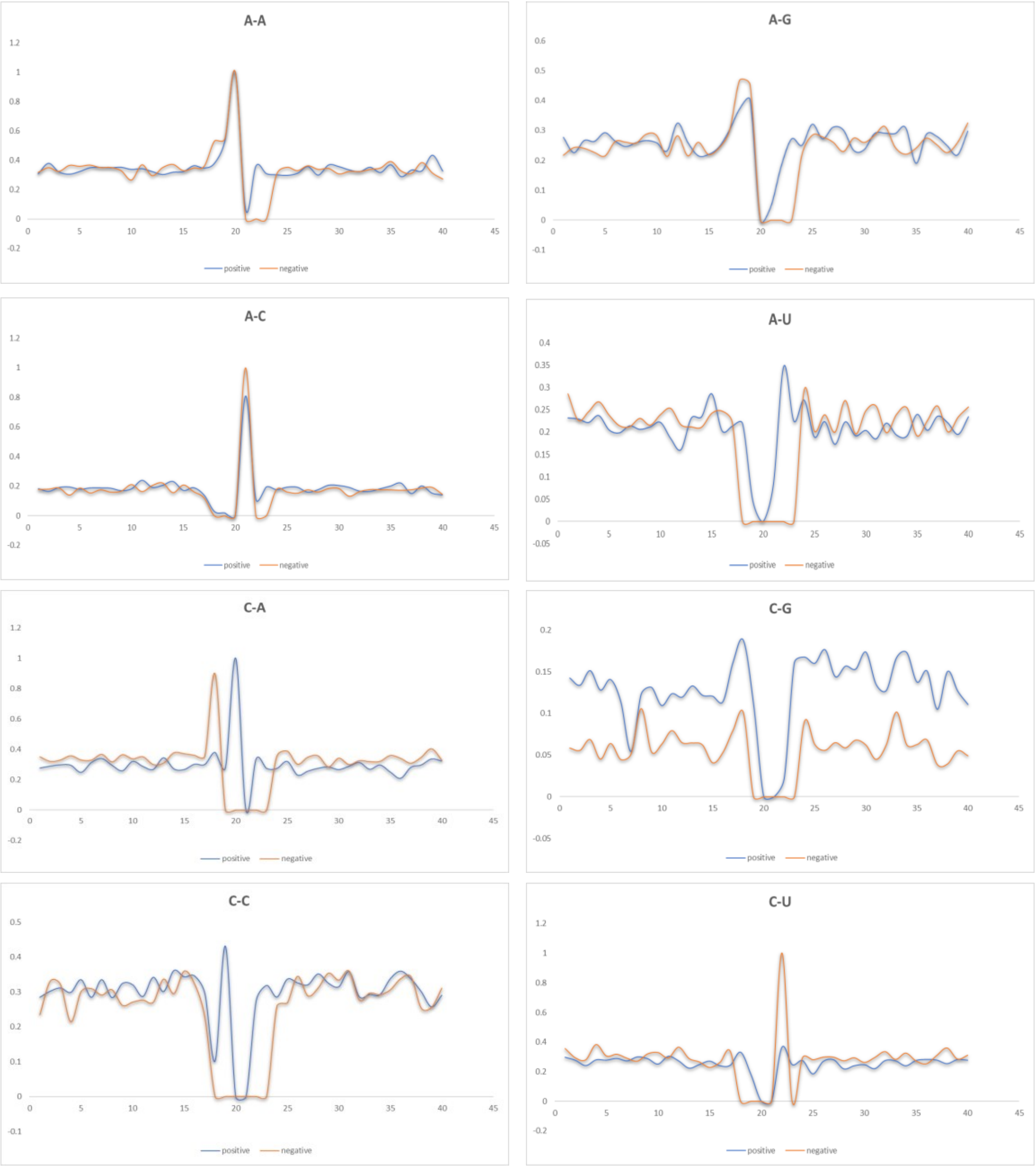
A-A
A-G
A-C
A-U
C-A
C-G
C-C
C-U
positive
negative

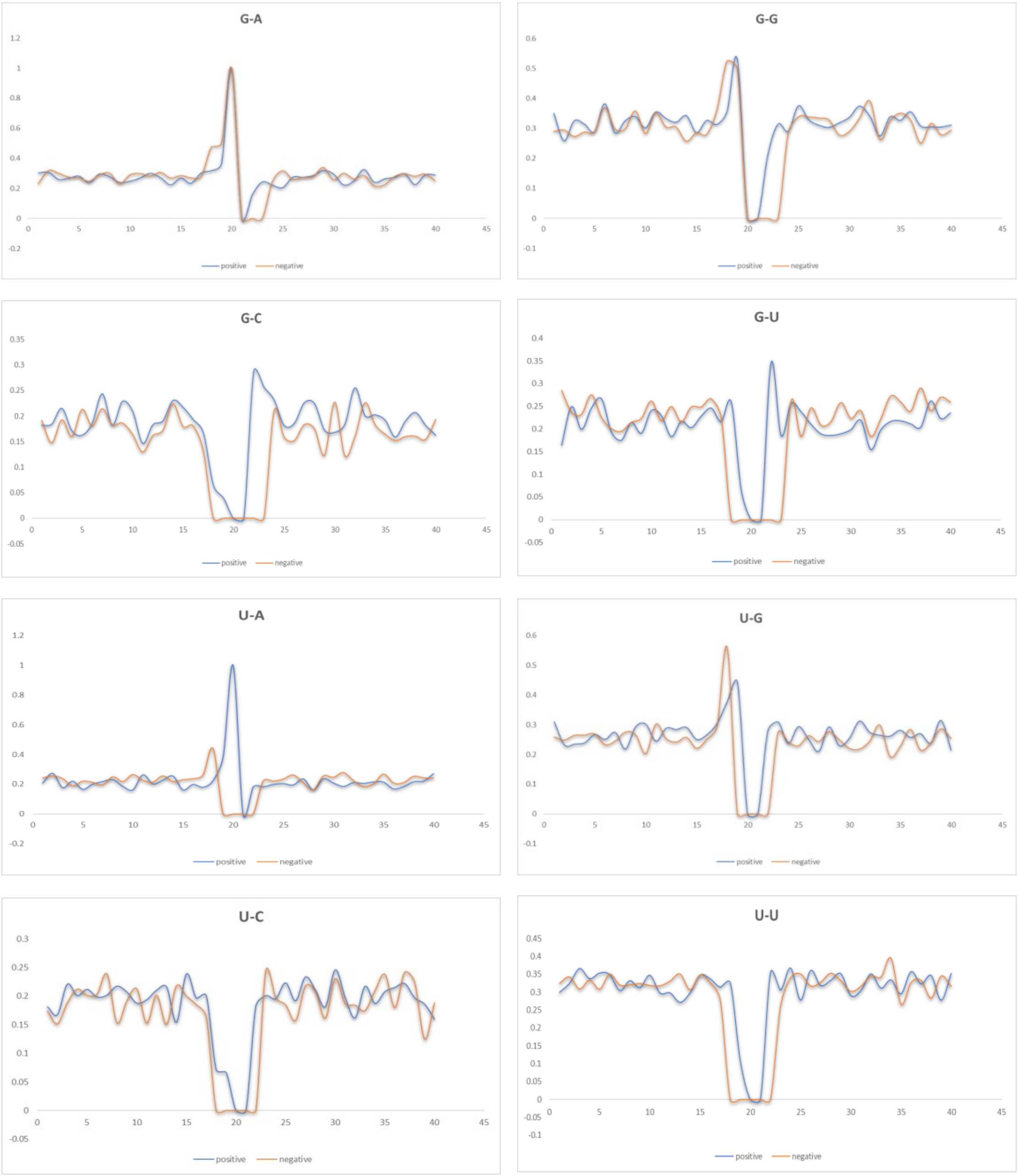

Figure S3 First-order transition probabilities of Homo sapiens data between adjacent nucleotides at all sites. The blue and bred lines represent the m6A and non-m6A sequences, respectively.
