# Supplementary material for: Identifying RNA N6-Methyladenine Sites in Three Species Based on a Markov Model
Source: Front Genet. 2021 Mar 19;12:650803. doi: 10.3389/fgene.2021.650803 (PMC8017269; doi:10.3389/fgene.2021.650803)
Supplement: Supplementary file 1 [file Presentation_1.zip › Supplementary Figures S5 - S6.PDF]

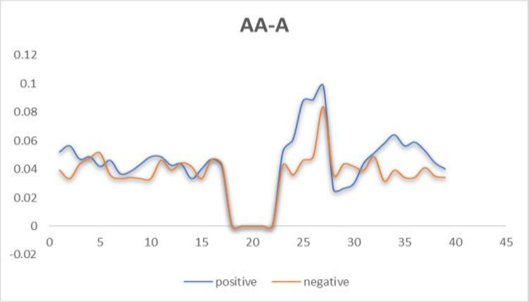
AA-A
0.12
0.1
0.08
0.06
0.04
0.02
0
-0.02
0 5 10 15 20 25 30 35 40 45
positive
negative

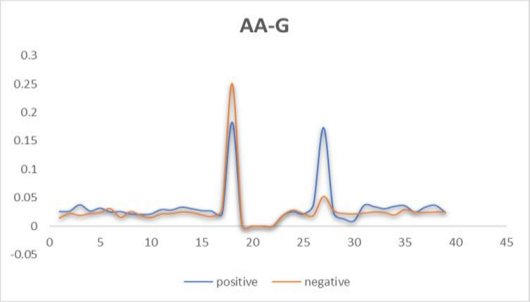
AA-G
0.3
0.25
0.2
0.15
0.1
0.05
0
-0.05
0 5 10 15 20 25 30 35 40 45
positive
negative

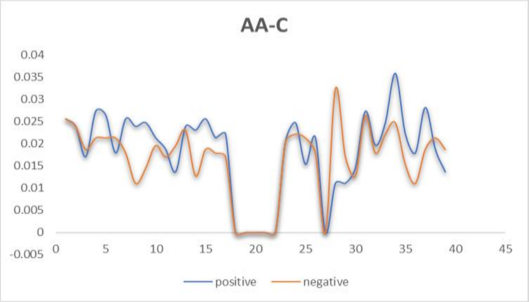
AA-C
0.04
0.035
0.03
0.025
0.02
0.015
0.01
0.005
0
-0.005
0 5 10 15 20 25 30 35 40 45
positive
negative

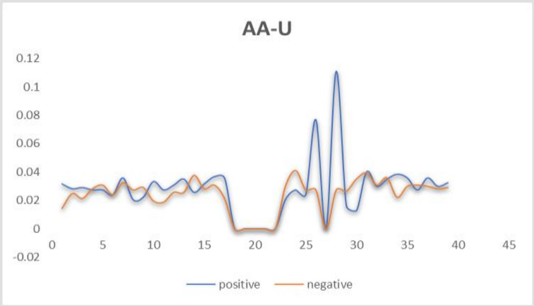
AA-U
0.12
0.1
0.08
0.06
0.04
0.02
0
-0.02
0 5 10 15 20 25 30 35 40 45
positive
negative

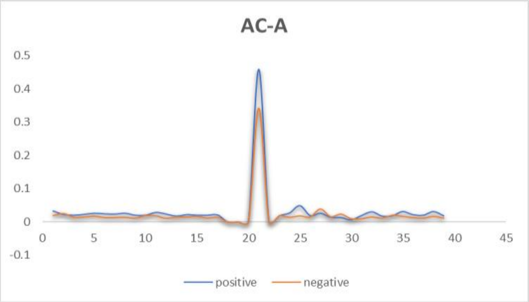
AC-A
0.5
0.4
0.3
0.2
0.1
0
-0.1
0 5 10 15 20 25 30 35 40 45
positive
negative

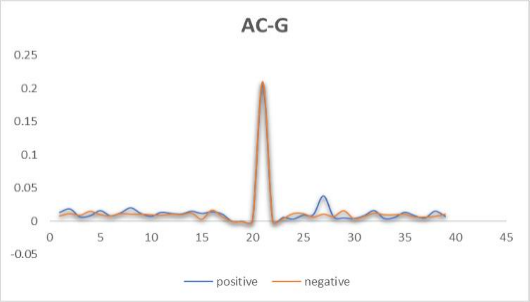
AC-G
0.25
0.2
0.15
0.1
0.05
0
-0.05
0 5 10 15 20 25 30 35 40 45
positive
negative

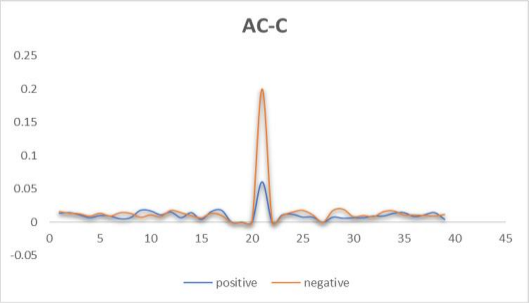
AC-C
0.25
0.2
0.15
0.1
0.05
0
-0.05
0 5 10 15 20 25 30 35 40 45
positive
negative

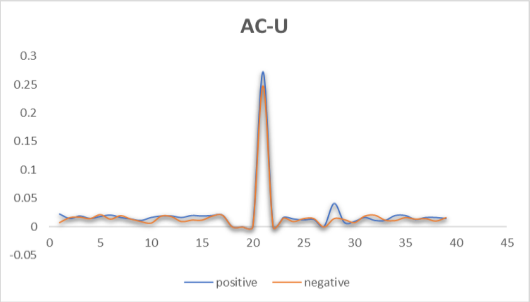
AC-U
0.3
0.25
0.2
0.15
0.1
0.05
0
-0.05
0 5 10 15 20 25 30 35 40 45
positive
negative

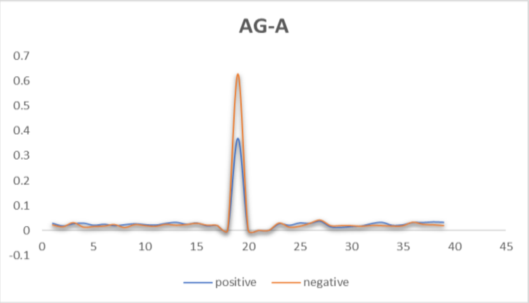
AG-A
0.7
0.6
0.5
0.4
0.3
0.2
0.1
0
-0.1
0 5 10 15 20 25 30 35 40 45
positive
negative

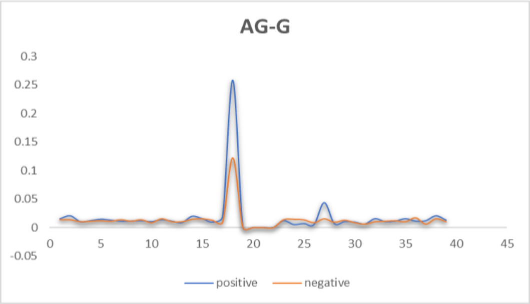
AG-G
0.3
0.25
0.2
0.15
0.1
0.05
0
-0.05
0 5 10 15 20 25 30 35 40 45
positive
negative

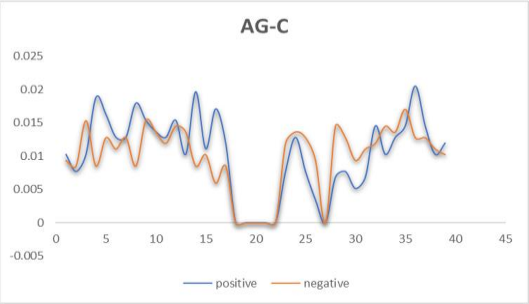
AG-C
0.025
0.02
0.015
0.01
0.005
0
-0.005
0 5 10 15 20 25 30 35 40 45
positive
negative

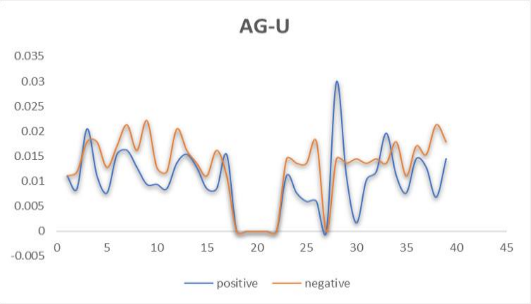
AG-U
0.035
0.03
0.025
0.02
0.015
0.01
0.005
0
-0.005
0 5 10 15 20 25 30 35 40 45
positive
negative

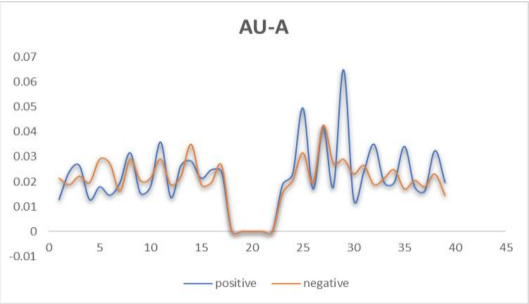
AU-A
0.07
0.06
0.05
0.04
0.03
0.02
0.01
0
-0.01
0 5 10 15 20 25 30 35 40 45
positive
negative

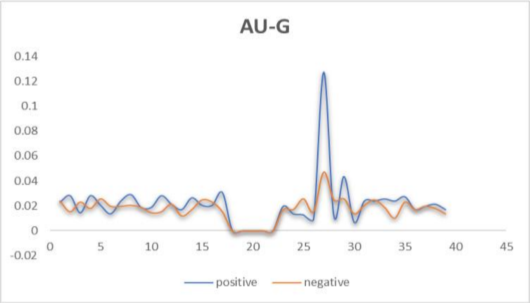
AU-G
0.14
0.12
0.1
0.08
0.06
0.04
0.02
0
-0.02
0 5 10 15 20 25 30 35 40 45
positive
negative

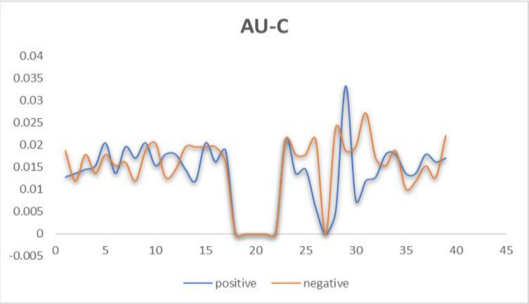
AU-C
0.04
0.035
0.03
0.025
0.02
0.015
0.01
0.005
0
-0.005
0 5 10 15 20 25 30 35 40 45
positive
negative

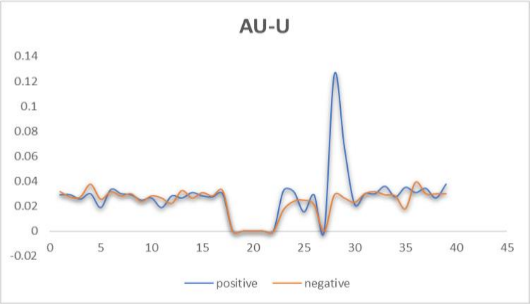
AU-U
0.14
0.12
0.1
0.08
0.06
0.04
0.02
0
-0.02
0 5 10 15 20 25 30 35 40 45
positive
negative

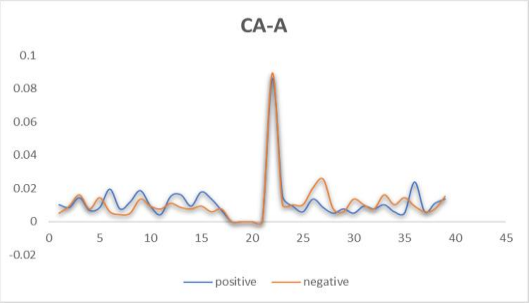
CA-A
0.1
0.08
0.06
0.04
0.02
0
-0.02
0 5 10 15 20 25 30 35 40 45
positive
negative

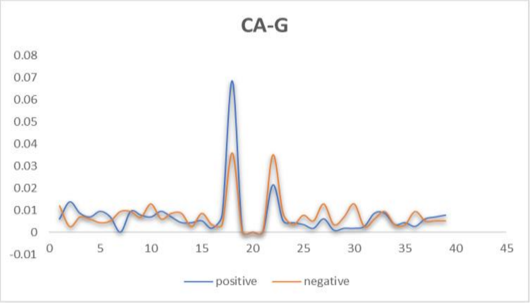
CA-G
0.08
0.07
0.06
0.05
0.04
0.03
0.02
0.01
0
-0.01
0 5 10 15 20 25 30 35 40 45
positive
negative

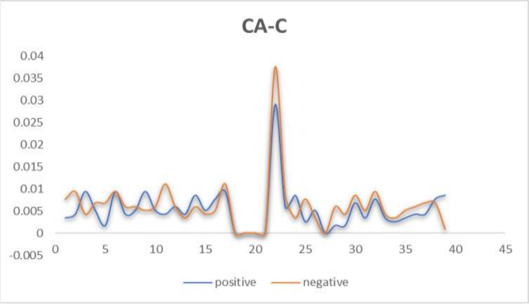
CA-C
0.04
0.035
0.03
0.025
0.02
0.015
0.01
0.005
0
-0.005
0 5 10 15 20 25 30 35 40 45
positive
negative

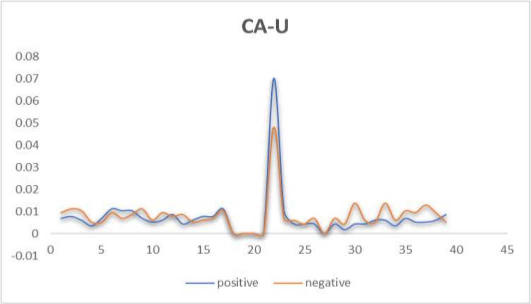
CA-U
0.08
0.07
0.06
0.05
0.04
0.03
0.02
0.01
0
-0.01
0 5 10 15 20 25 30 35 40 45
positive
negative

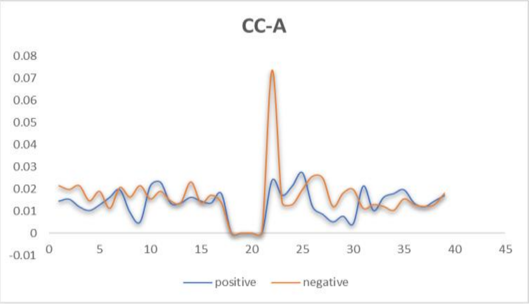
CC-A
0.08
0.07
0.06
0.05
0.04
0.03
0.02
0.01
0
-0.01
0 5 10 15 20 25 30 35 40 45
positive
negative

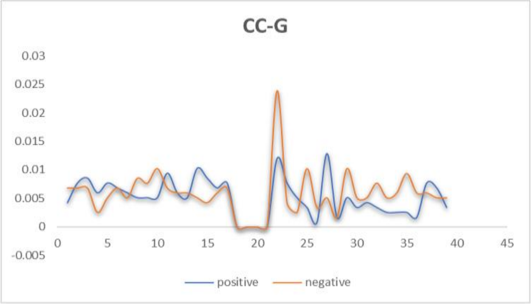
CC-G
0.03
0.025
0.02
0.015
0.01
0.005
0
-0.005
0 5 10 15 20 25 30 35 40 45
positive
negative

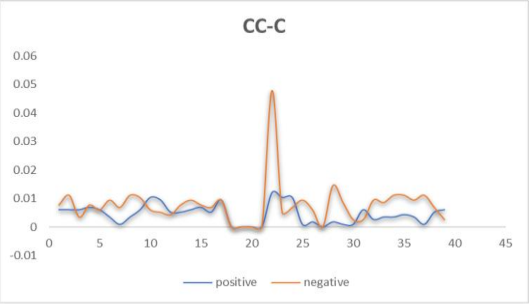
CC-C
0.06
0.05
0.04
0.03
0.02
0.01
0
-0.01
0 5 10 15 20 25 30 35 40 45
positive
negative

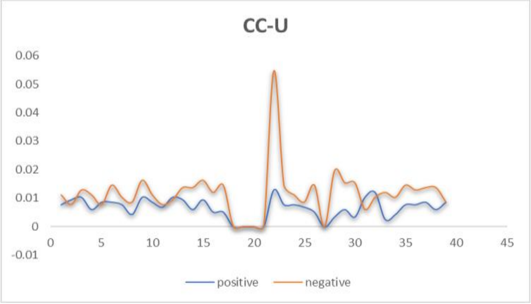
CC-U
0.06
0.05
0.04
0.03
0.02
0.01
0
-0.01
0 5 10 15 20 25 30 35 40 45
positive
negative

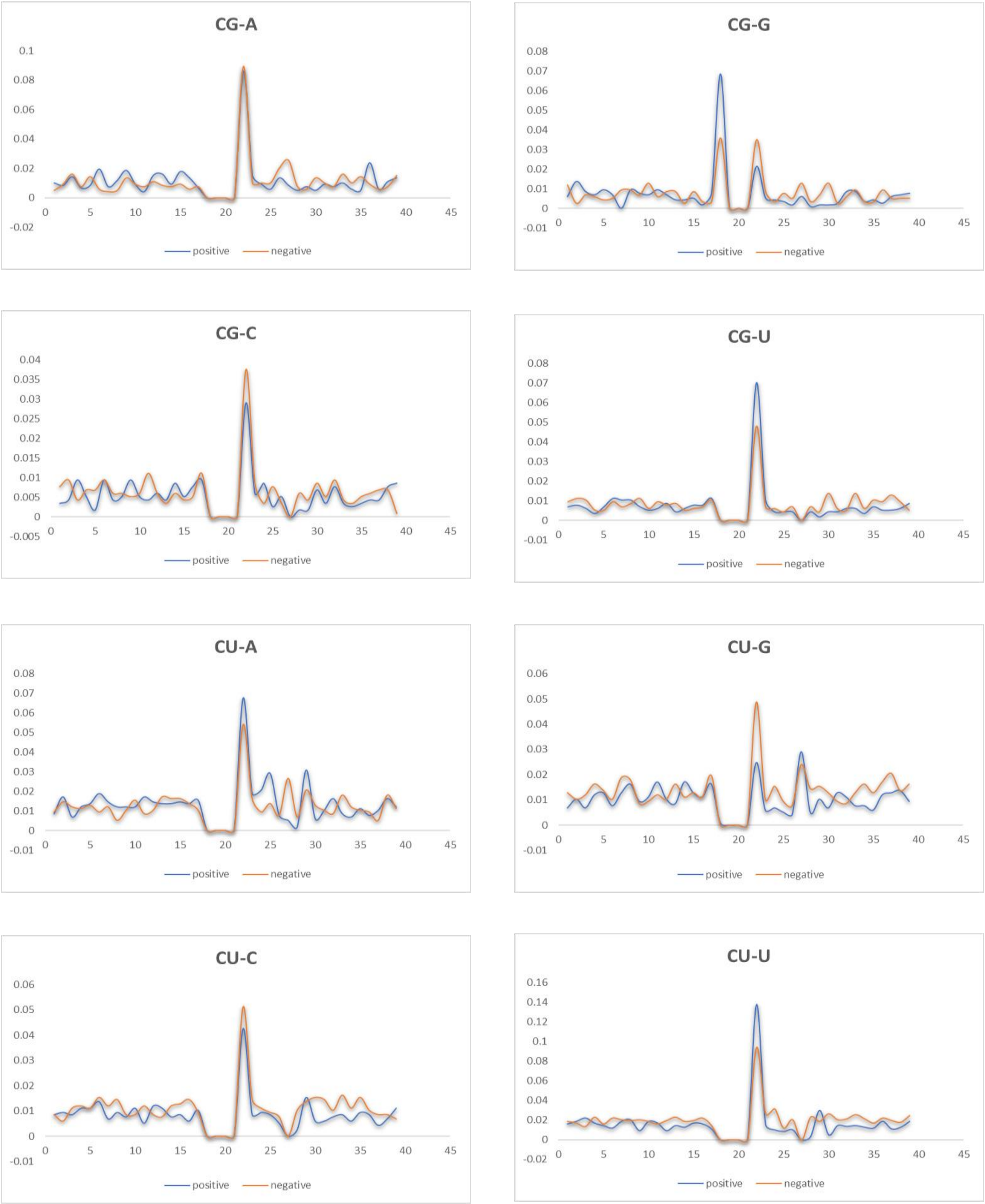
CG-A
0.1
0.08
0.06
0.04
0.02
0
-0.02
0 5 10 15 20 25 30 35 40 45
positive negative
CG-G
0.08
0.07
0.06
0.05
0.04
0.03
0.02
0.01
0
-0.01
0 5 10 15 20 25 30 35 40 45
positive negative
CG-C
0.04
0.035
0.03
0.025
0.02
0.015
0.01
0.005
0
-0.005
0 5 10 15 20 25 30 35 40 45
positive negative
CG-U
0.08
0.07
0.06
0.05
0.04
0.03
0.02
0.01
0
-0.01
0 5 10 15 20 25 30 35 40 45
positive negative
CU-A
0.08
0.07
0.06
0.05
0.04
0.03
0.02
0.01
0
-0.01
0 5 10 15 20 25 30 35 40 45
positive negative
CU-G
0.06
0.05
0.04
0.03
0.02
0.01
0
-0.01
0 5 10 15 20 25 30 35 40 45
positive negative
CU-C
0.06
0.05
0.04
0.03
0.02
0.01
0
-0.01
0 5 10 15 20 25 30 35 40 45
positive negative
CU-U
0.16
0.14
0.12
0.1
0.08
0.06
0.04
0.02
0
-0.02
0 5 10 15 20 25 30 35 40 45
positive negative

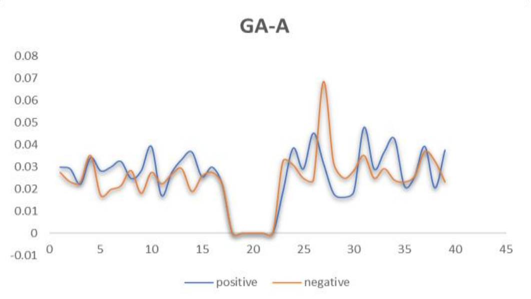
GA-A
0.08 0.07 0.06 0.05 0.04 0.03 0.02 0.01 0 -0.01
0 5 10 15 20 25 30 35 40 45
positive negative

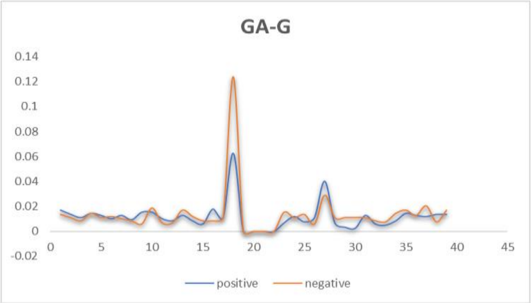
GA-G
0.14 0.12 0.1 0.08 0.06 0.04 0.02 0 -0.02
0 5 10 15 20 25 30 35 40 45
positive negative

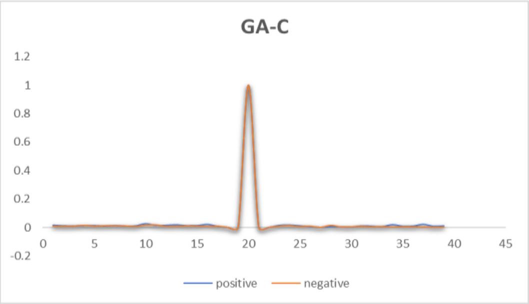
GA-C
1.2 1 0.8 0.6 0.4 0.2 0 -0.2
0 5 10 15 20 25 30 35 40 45
positive negative

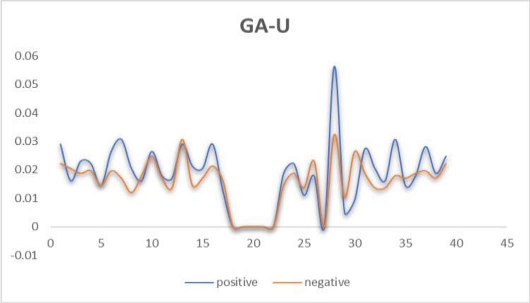
GA-U
0.06 0.05 0.04 0.03 0.02 0.01 0 -0.01
0 5 10 15 20 25 30 35 40 45
positive negative

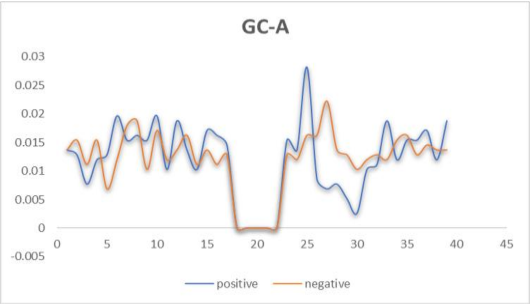
GC-A
0.03 0.025 0.02 0.015 0.01 0.005 0 -0.005
0 5 10 15 20 25 30 35 40 45
positive negative

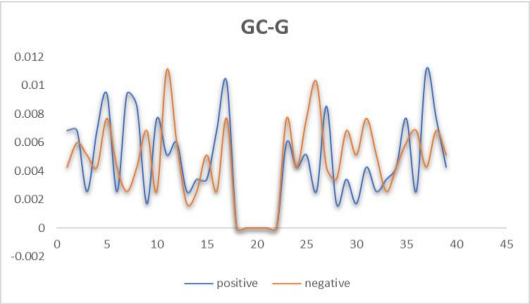
GC-G
0.012 0.01 0.008 0.006 0.004 0.002 0 -0.002
0 5 10 15 20 25 30 35 40 45
positive negative

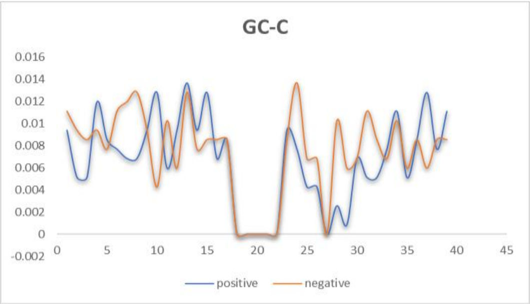
GC-C
0.016 0.014 0.012 0.01 0.008 0.006 0.004 0.002 0 -0.002
0 5 10 15 20 25 30 35 40 45
positive negative

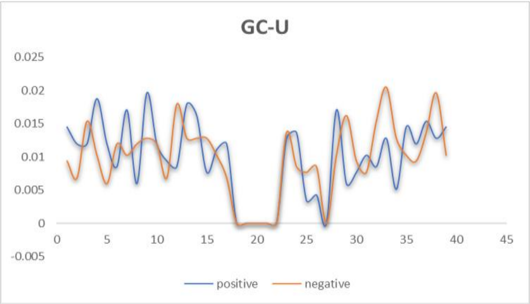
GC-U
0.025 0.02 0.015 0.01 0.005 0 -0.005
0 5 10 15 20 25 30 35 40 45
positive negative

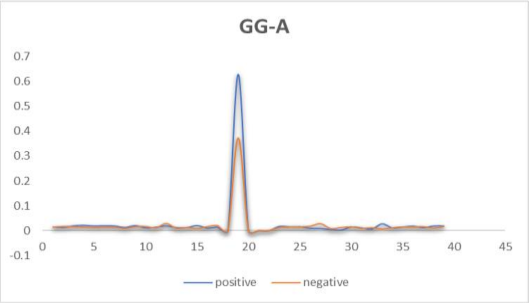
GG-A
0.7
0.6
0.5
0.4
0.3
0.2
0.1
0
-0.1
0 5 10 15 20 25 30 35 40 45
positive
negative

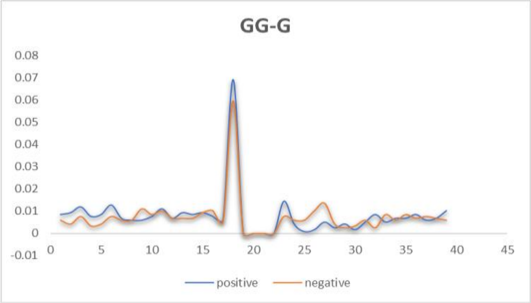
GG-G
0.08
0.07
0.06
0.05
0.04
0.03
0.02
0.01
0
-0.01
0 5 10 15 20 25 30 35 40 45
positive
negative

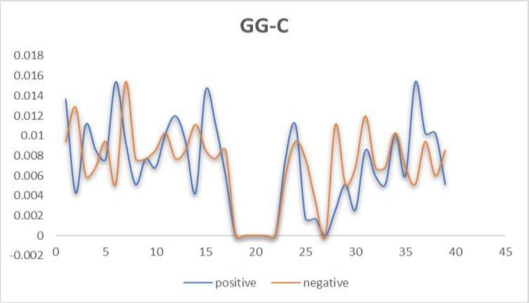
GG-C
0.018
0.016
0.014
0.012
0.01
0.008
0.006
0.004
0.002
0
-0.002
0 5 10 15 20 25 30 35 40 45
positive
negative

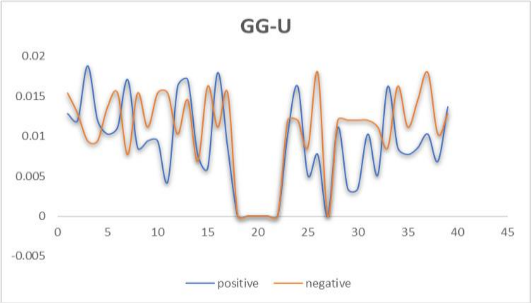
GG-U
0.02
0.015
0.01
0.005
0
-0.005
0 5 10 15 20 25 30 35 40 45
positive
negative

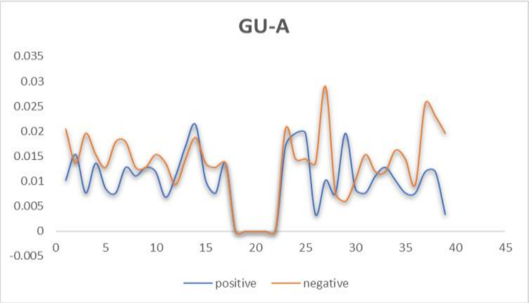
GU-A
0.035
0.03
0.025
0.02
0.015
0.01
0.005
0
-0.005
0 5 10 15 20 25 30 35 40 45
positive
negative

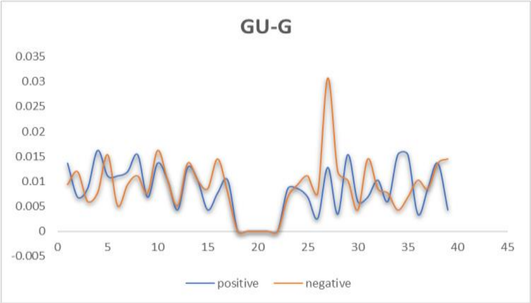
GU-G
0.035
0.03
0.025
0.02
0.015
0.01
0.005
0
-0.005
0 5 10 15 20 25 30 35 40 45
positive
negative

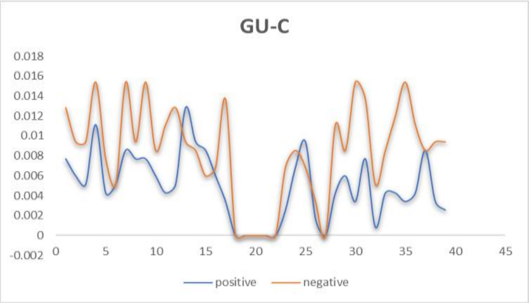
GU-C
0.018
0.016
0.014
0.012
0.01
0.008
0.006
0.004
0.002
0
-0.002
0 5 10 15 20 25 30 35 40 45
positive
negative

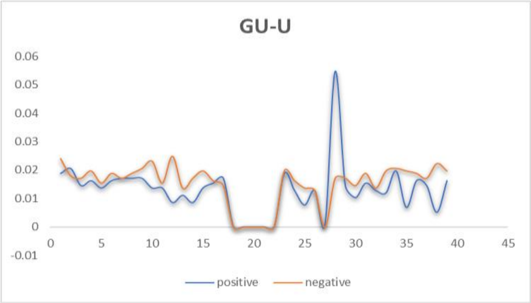
GU-U
0.06
0.05
0.04
0.03
0.02
0.01
0
-0.01
0 5 10 15 20 25 30 35 40 45
positive
negative

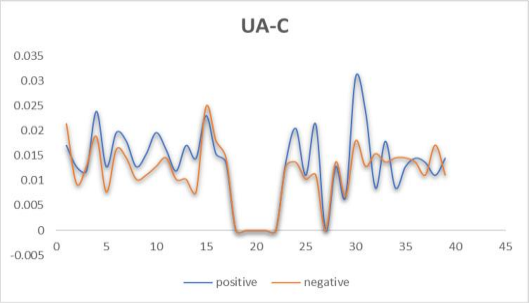
UA-C
positive
negative

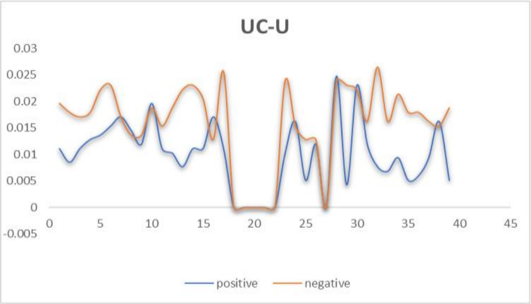
UC-U
positive
negative

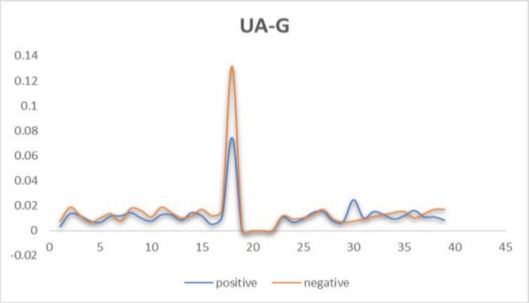
UA-G
positive
negative

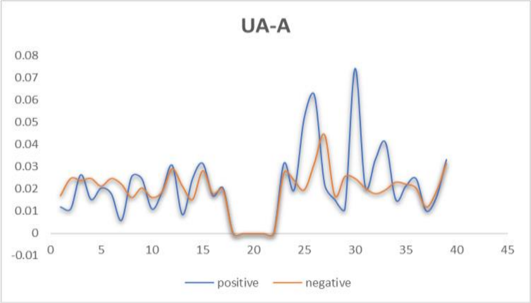
UA-A
positive
negative

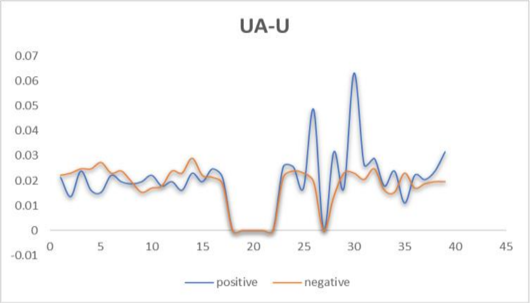
UA-U
positive
negative

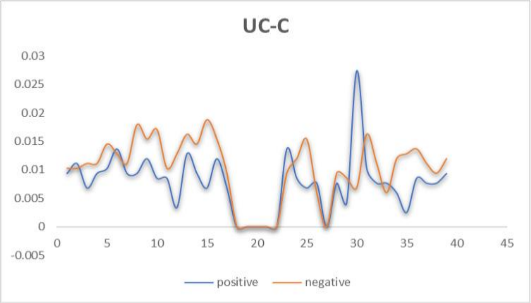
UC-C
positive
negative

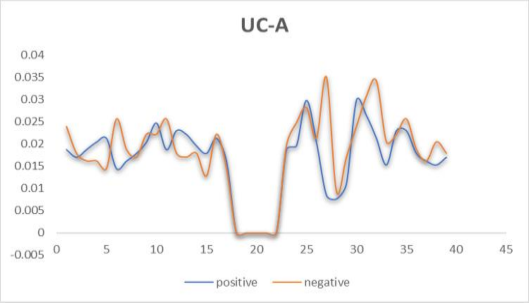
UC-A
positive
negative

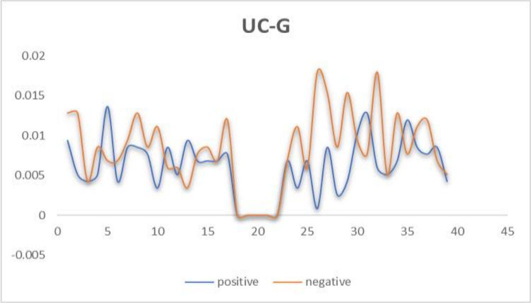
UC-G
positive
negative

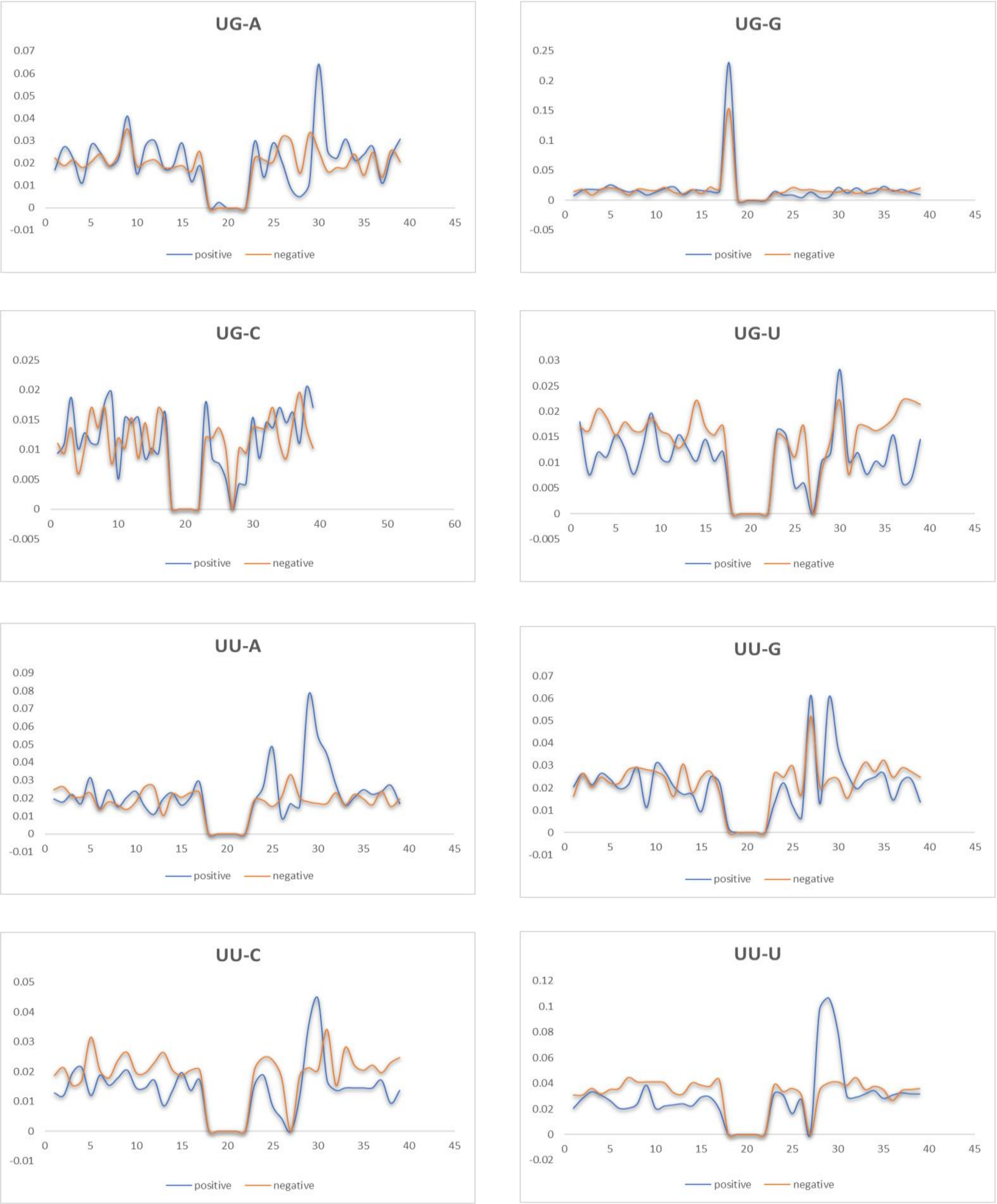

Figure S4 Second-order transition probabilities of Saccharomyces data between adjacent nucleotides at all sites. The blue and red lines represent the m6A and non-m6A sequences, respectively.

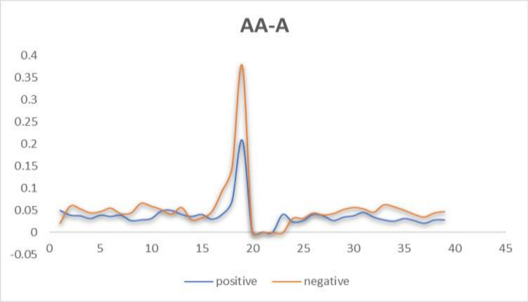
AA-A
0.4 0.35 0.3 0.25 0.2 0.15 0.1 0.05 0 -0.05
0 5 10 15 20 25 30 35 40 45
positive negative

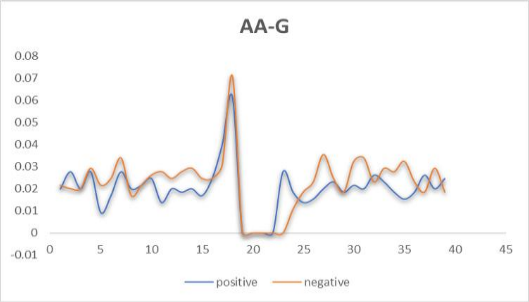
AA-G
0.08 0.07 0.06 0.05 0.04 0.03 0.02 0.01 0 -0.01
0 5 10 15 20 25 30 35 40 45
positive negative

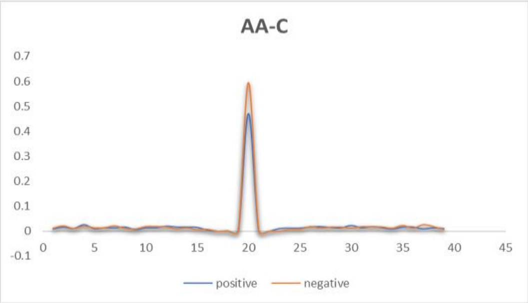
AA-C
0.7 0.6 0.5 0.4 0.3 0.2 0.1 0 -0.1
0 5 10 15 20 25 30 35 40 45
positive negative

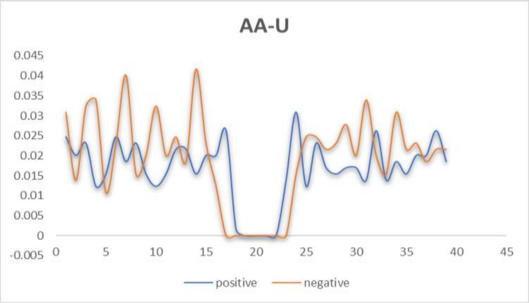
AA-U
0.045 0.04 0.035 0.03 0.025 0.02 0.015 0.01 0.005 0 -0.005
0 5 10 15 20 25 30 35 40 45
positive negative

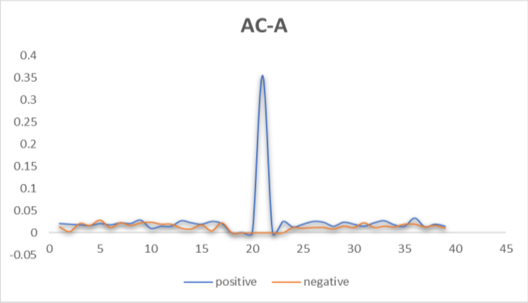
AC-A
0.4 0.35 0.3 0.25 0.2 0.15 0.1 0.05 0 -0.05
0 5 10 15 20 25 30 35 40 45
positive negative

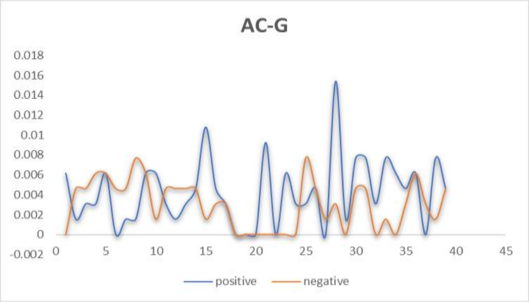
AC-G
0.018 0.016 0.014 0.012 0.01 0.008 0.006 0.004 0.002 0 -0.002
0 5 10 15 20 25 30 35 40 45
positive negative

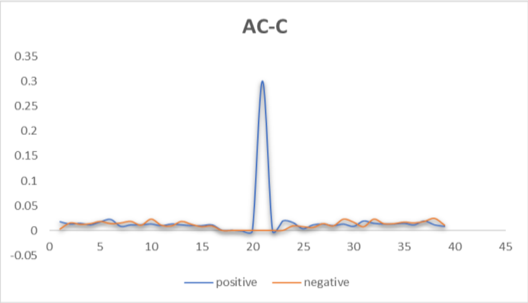
AC-C
0.35 0.3 0.25 0.2 0.15 0.1 0.05 0 -0.05
0 5 10 15 20 25 30 35 40 45
positive negative

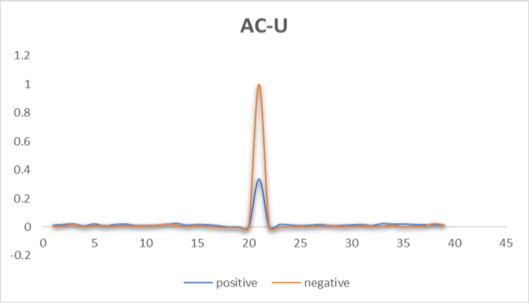
AC-U
1.2 1 0.8 0.6 0.4 0.2 0 -0.2
0 5 10 15 20 25 30 35 40 45
positive negative

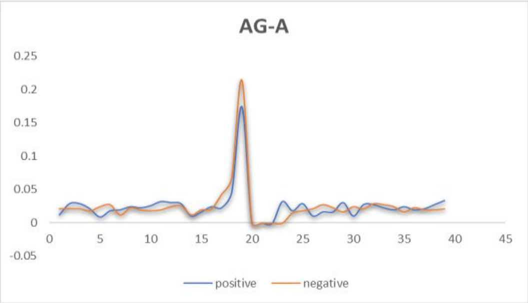
AG-A
0.25 0.2 0.15 0.1 0.05 0 -0.05
0 5 10 15 20 25 30 35 40 45
positive negative

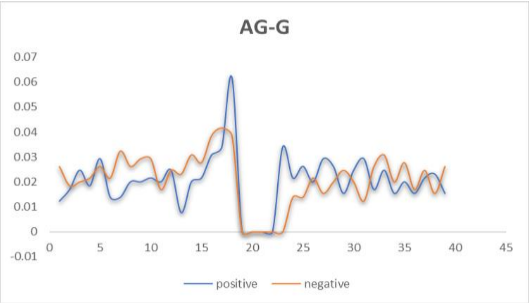
AG-G
0.07 0.06 0.05 0.04 0.03 0.02 0.01 0 -0.01
0 5 10 15 20 25 30 35 40 45
positive negative

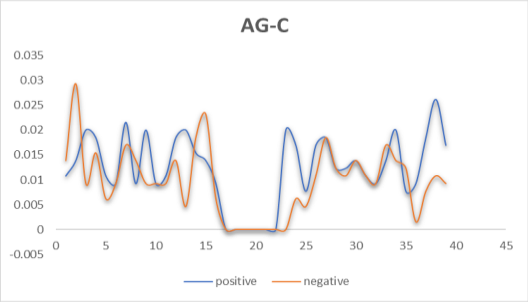
AG-C
0.035
0.03
0.025
0.02
0.015
0.01
0.005
0
-0.005
0 5 10 15 20 25 30 35 40 45
positive
negative

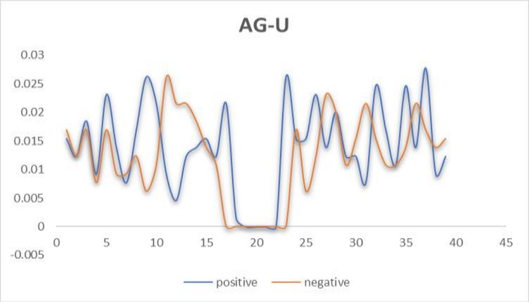
AG-U
0.03
0.025
0.02
0.015
0.01
0.005
0
-0.005
0 5 10 15 20 25 30 35 40 45
positive
negative

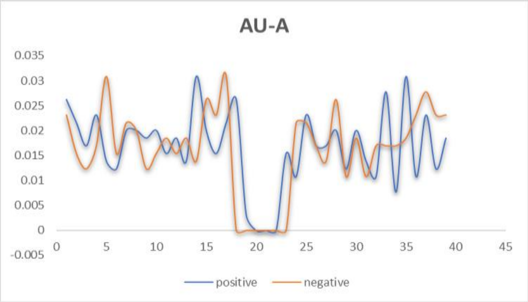
AU-A
0.035
0.03
0.025
0.02
0.015
0.01
0.005
0
-0.005
0 5 10 15 20 25 30 35 40 45
positive
negative

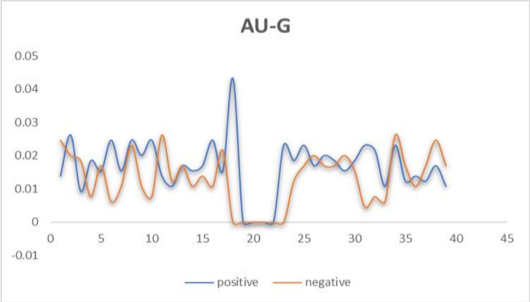
AU-G
0.05
0.04
0.03
0.02
0.01
0
-0.01
0 5 10 15 20 25 30 35 40 45
positive
negative

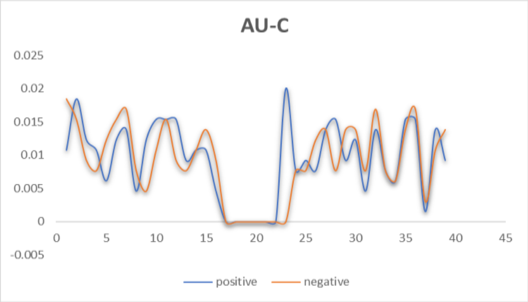
AU-C
0.025
0.02
0.015
0.01
0.005
0
-0.005
0 5 10 15 20 25 30 35 40 45
positive
negative

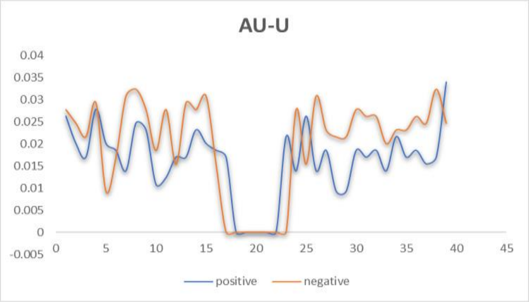
AU-U
0.04
0.035
0.03
0.025
0.02
0.015
0.01
0.005
0
-0.005
0 5 10 15 20 25 30 35 40 45
positive
negative

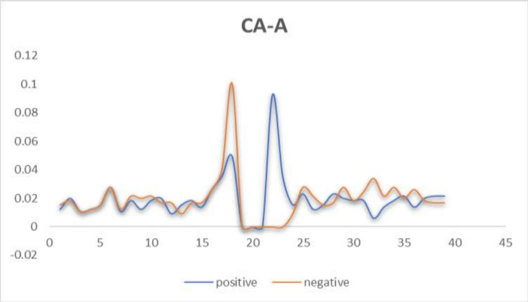
CA-A
0.12
0.1
0.08
0.06
0.04
0.02
0
-0.02
0 5 10 15 20 25 30 35 40 45
positive
negative

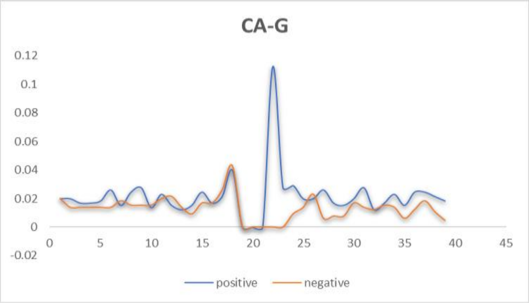
CA-G
0.12
0.1
0.08
0.06
0.04
0.02
0
-0.02
0 5 10 15 20 25 30 35 40 45
positive
negative

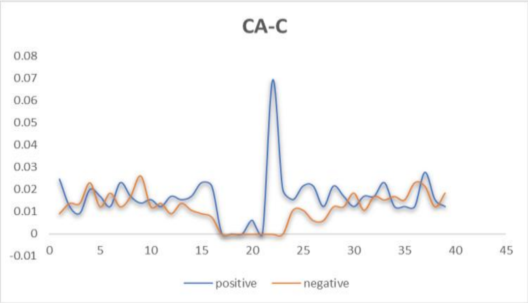
CA-C
0.08
0.07
0.06
0.05
0.04
0.03
0.02
0.01
0
-0.01
0 5 10 15 20 25 30 35 40 45
positive
negative

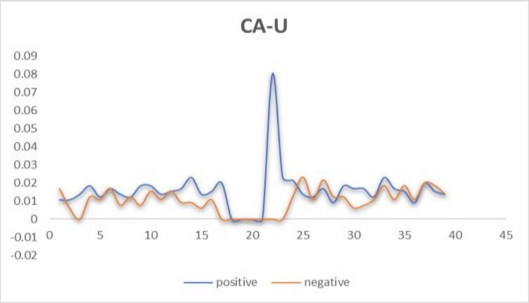
CA-U
0.09
0.08
0.07
0.06
0.05
0.04
0.03
0.02
0.01
0
-0.01
-0.02
0 5 10 15 20 25 30 35 40 45
positive
negative

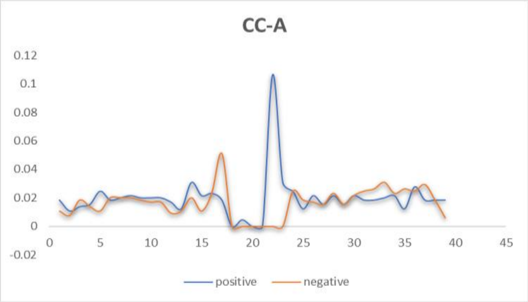
CC-A
positive
negative

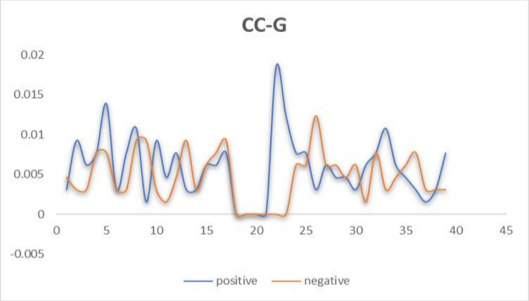
CC-G
positive
negative

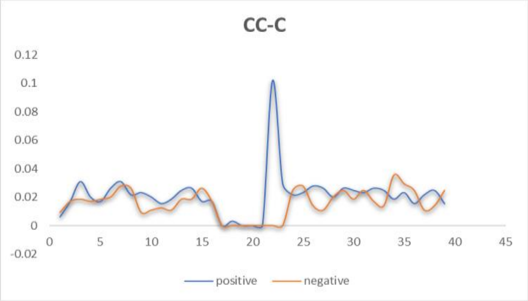
CC-C
positive
negative

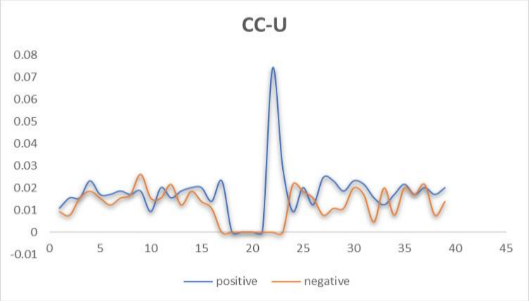
CC-U
positive
negative

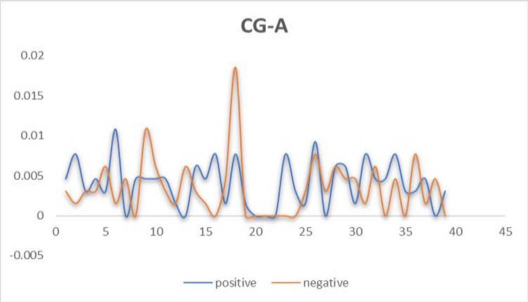
CG-A
positive
negative

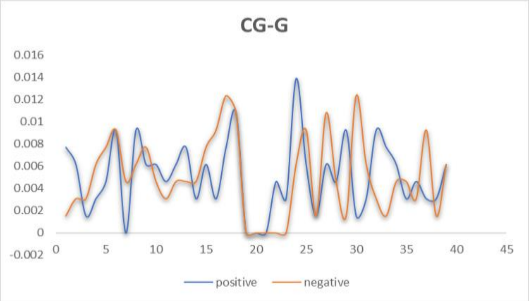
CG-G
positive
negative

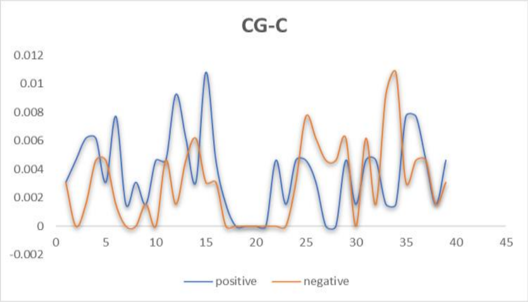
CG-C
positive
negative

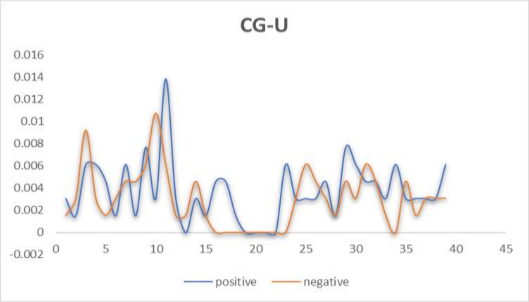
CG-U
positive
negative

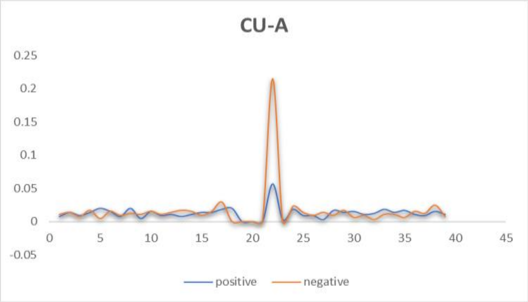
CU-A
positive
negative

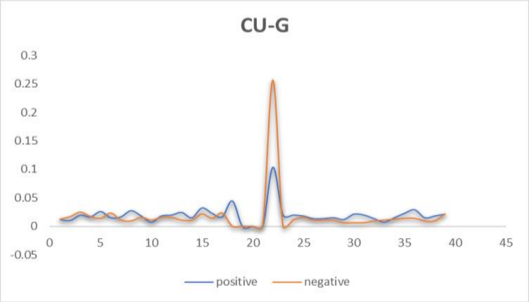
CU-G
positive
negative

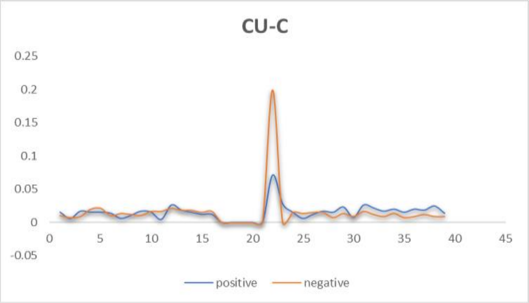
CU-C
positive
negative

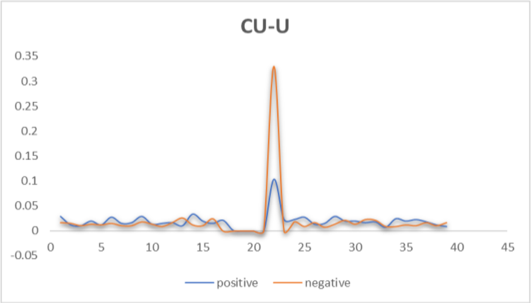
CU-U
positive
negative

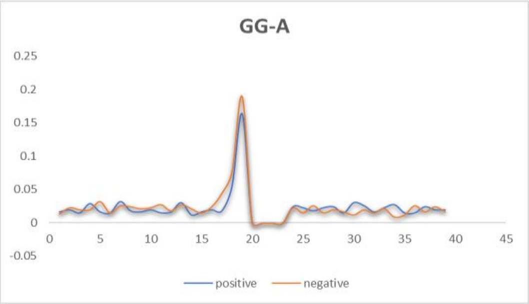
GG-A
positive
negative

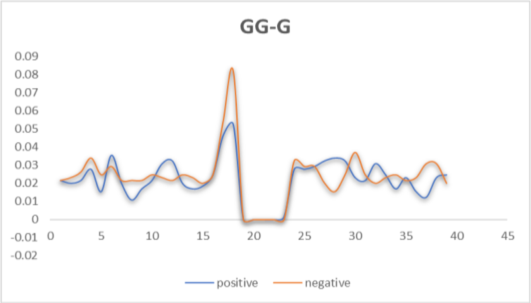
GG-G
positive
negative

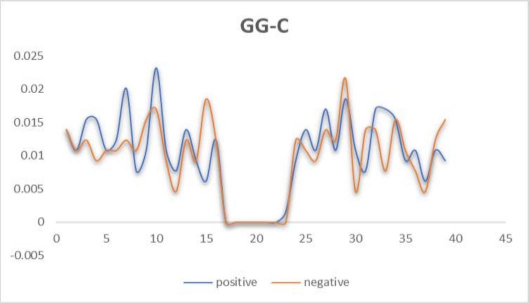
GG-C
positive
negative

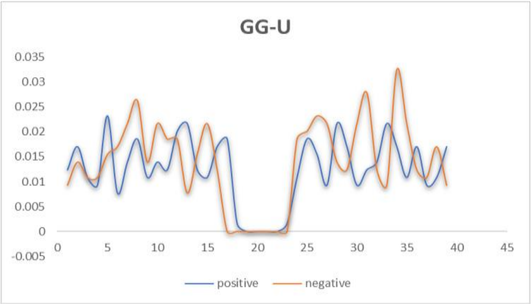
GG-U
positive
negative

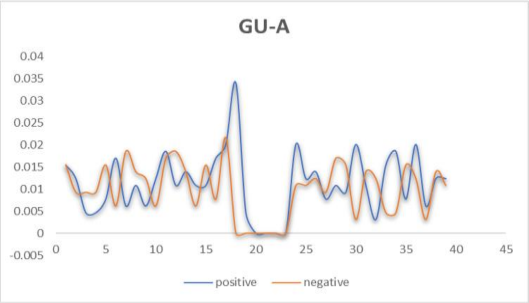
GU-A
positive
negative

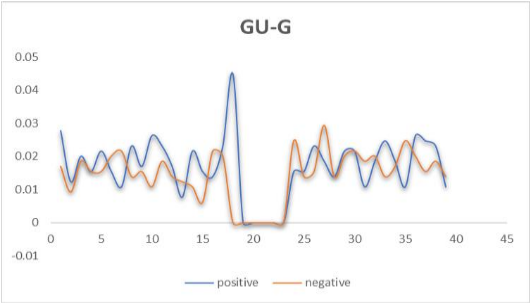
GU-G
positive
negative

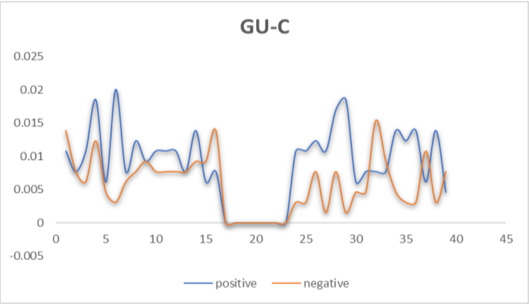
GU-C
positive
negative

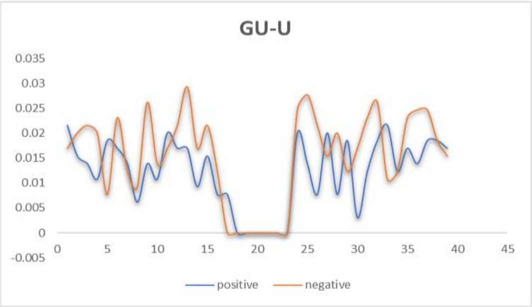
GU-U
positive
negative

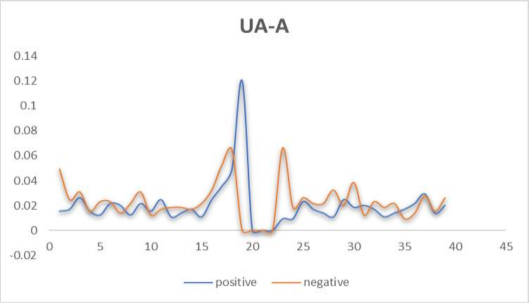
UA-A
0.14
0.12
0.1
0.08
0.06
0.04
0.02
0
-0.02
0 5 10 15 20 25 30 35 40 45
positive
negative

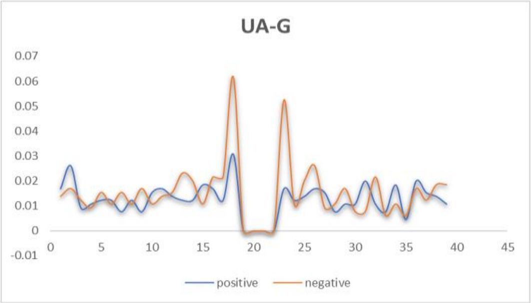
UA-G
0.07
0.06
0.05
0.04
0.03
0.02
0.01
0
-0.01
0 5 10 15 20 25 30 35 40 45
positive
negative

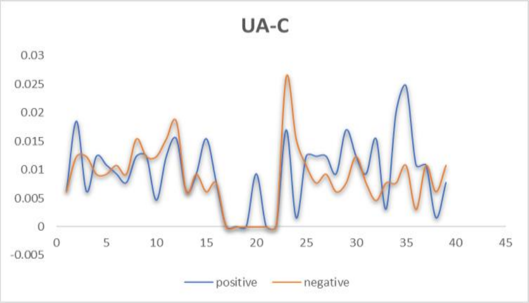
UA-C
0.03
0.025
0.02
0.015
0.01
0.005
0
-0.005
0 5 10 15 20 25 30 35 40 45
positive
negative

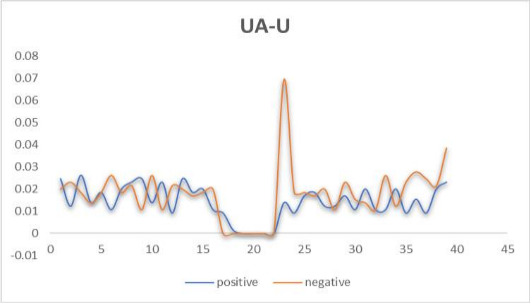
UA-U
0.08
0.07
0.06
0.05
0.04
0.03
0.02
0.01
0
-0.01
0 5 10 15 20 25 30 35 40 45
positive
negative

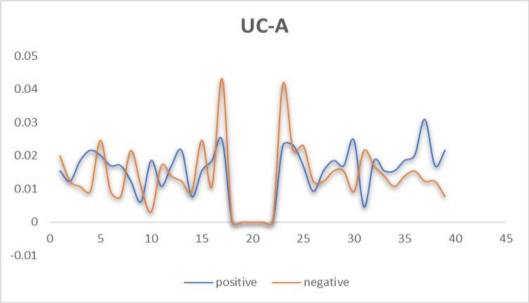
UC-A
0.05
0.04
0.03
0.02
0.01
0
-0.01
0 5 10 15 20 25 30 35 40 45
positive
negative

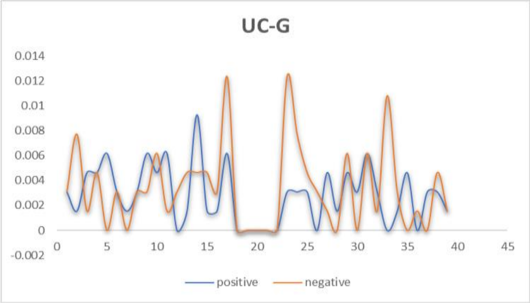
UC-G
0.014
0.012
0.01
0.008
0.006
0.004
0.002
0
-0.002
0 5 10 15 20 25 30 35 40 45
positive
negative

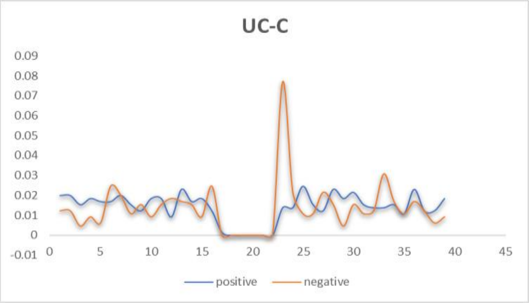
UC-C
0.09
0.08
0.07
0.06
0.05
0.04
0.03
0.02
0.01
0
-0.01
0 5 10 15 20 25 30 35 40 45
positive
negative

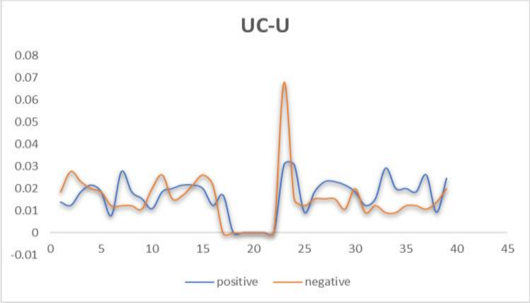
UC-U
0.08
0.07
0.06
0.05
0.04
0.03
0.02
0.01
0
-0.01
0 5 10 15 20 25 30 35 40 45
positive
negative

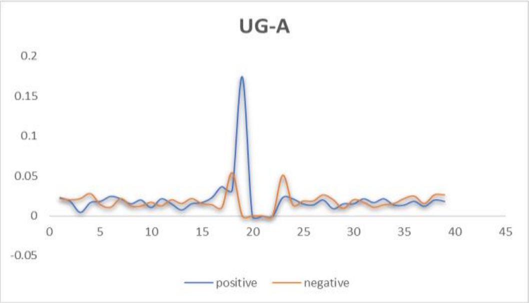
UG-A
0.2
0.15
0.1
0.05
0
-0.05
0 5 10 15 20 25 30 35 40 45
positive
negative

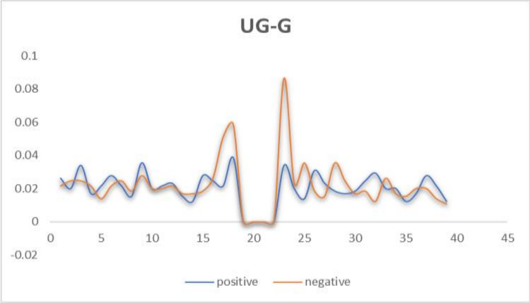
UG-G
0.1
0.08
0.06
0.04
0.02
0
-0.02
0 5 10 15 20 25 30 35 40 45
positive
negative

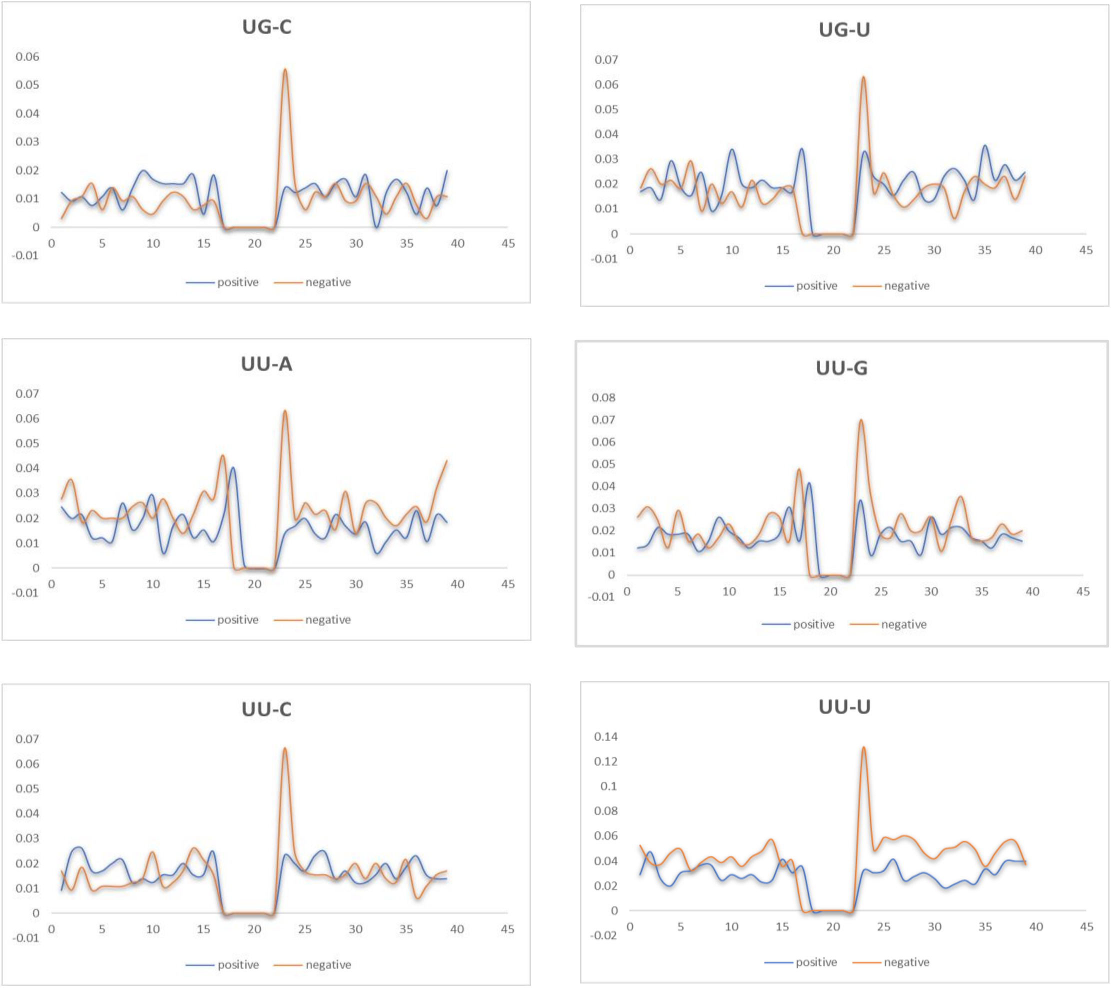

Figure S5 Second-order transition probabilities of Mouse data between adjacent nucleotides at all sites. The blue and red lines represent the m6A and non-m6A sequences, respectively.

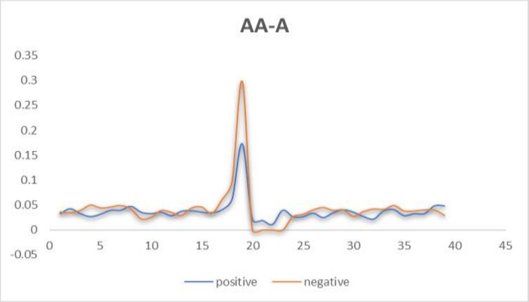
AA-A
positive
negative

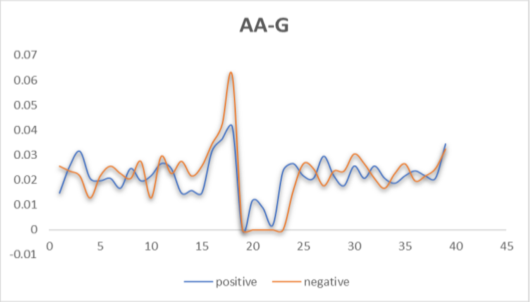
AA-G
positive
negative

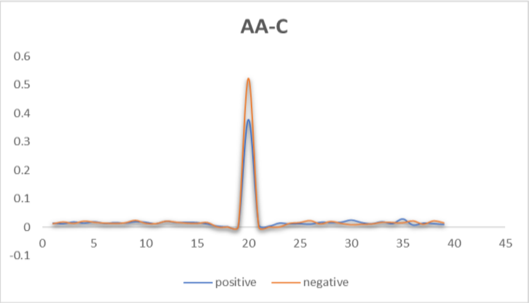
AA-C
positive
negative

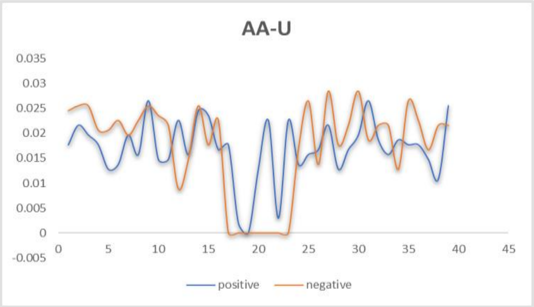
AA-U
positive
negative

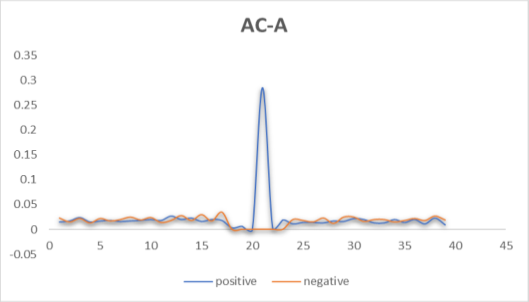
AC-A
positive
negative

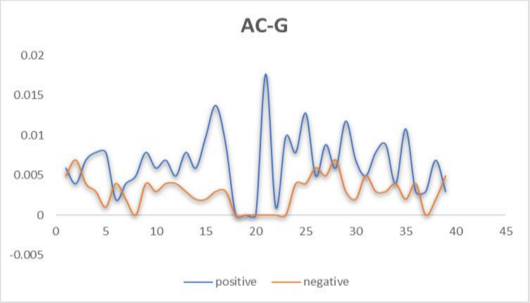
AC-G
positive
negative

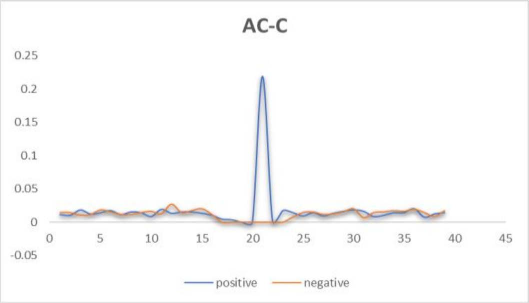
AC-C
positive
negative

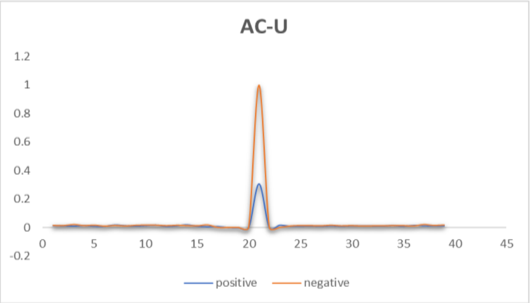
AC-U
positive
negative

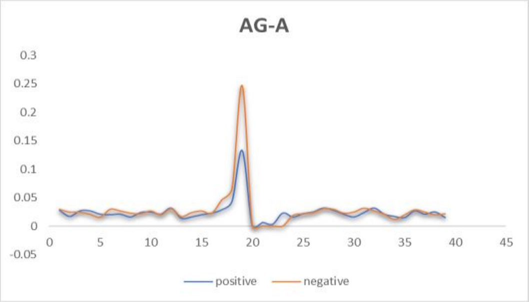
AG-A
positive
negative

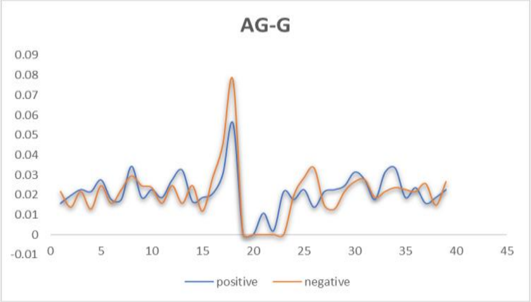
AG-G
positive
negative

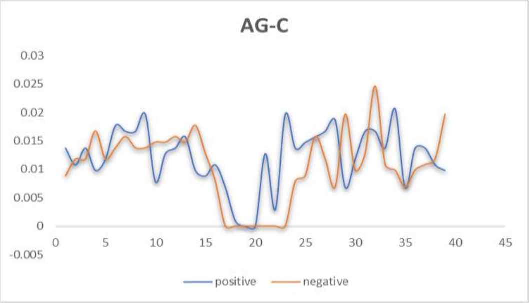
AG-C
positive
negative

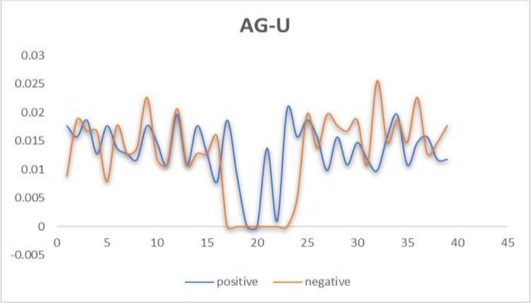
AG-U
positive
negative

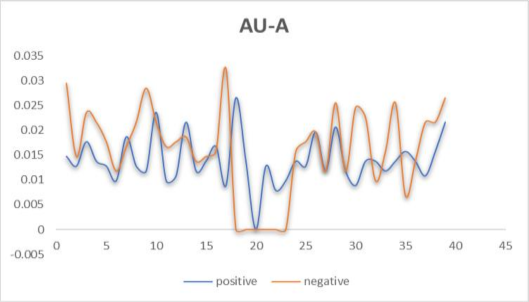
AU-A
positive
negative

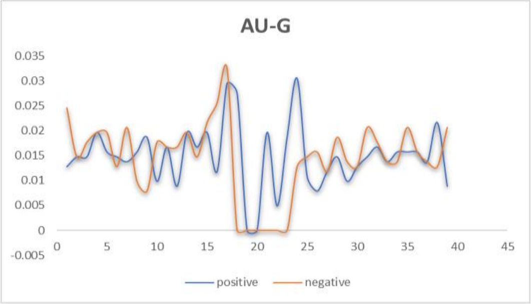
AU-G
positive
negative

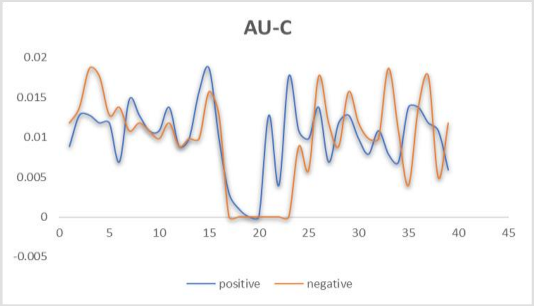
AU-C
positive
negative

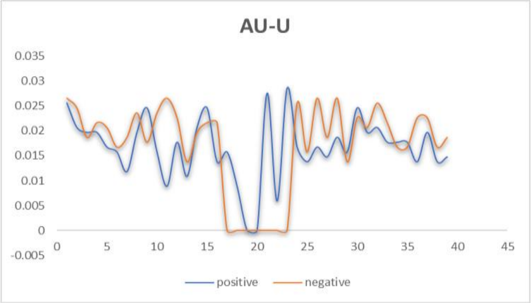
AU-U
positive
negative

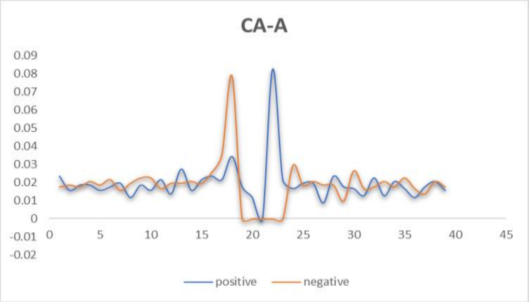
CA-A
0.09
0.08
0.07
0.06
0.05
0.04
0.03
0.02
0.01
0
-0.01
-0.02
0 5 10 15 20 25 30 35 40 45
positive
negative

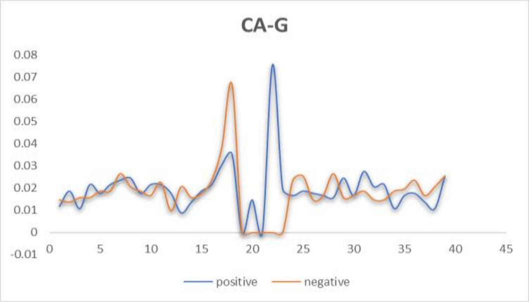
CA-G
0.08
0.07
0.06
0.05
0.04
0.03
0.02
0.01
0
-0.01
0 5 10 15 20 25 30 35 40 45
positive
negative

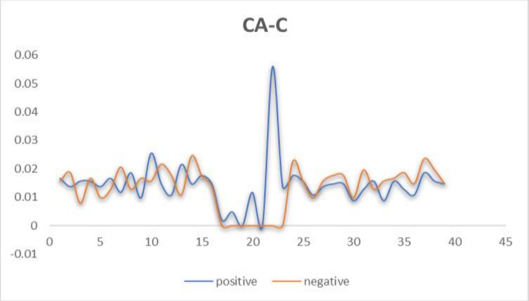
CA-C
0.06
0.05
0.04
0.03
0.02
0.01
0
-0.01
0 5 10 15 20 25 30 35 40 45
positive
negative

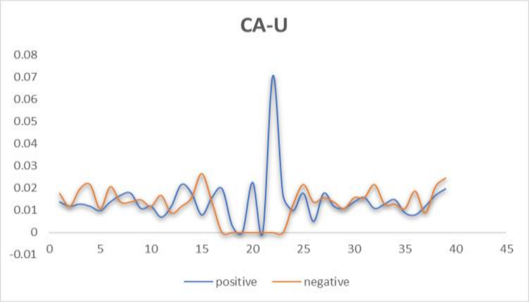
CA-U
0.08
0.07
0.06
0.05
0.04
0.03
0.02
0.01
0
-0.01
0 5 10 15 20 25 30 35 40 45
positive
negative

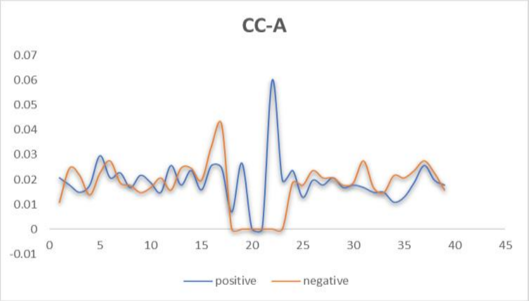
CC-A
0.07
0.06
0.05
0.04
0.03
0.02
0.01
0
-0.01
0 5 10 15 20 25 30 35 40 45
positive
negative

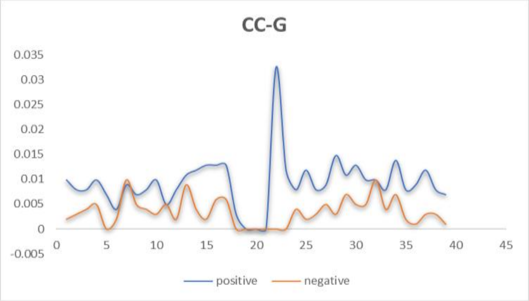
CC-G
0.035
0.03
0.025
0.02
0.015
0.01
0.005
0
-0.005
0 5 10 15 20 25 30 35 40 45
positive
negative

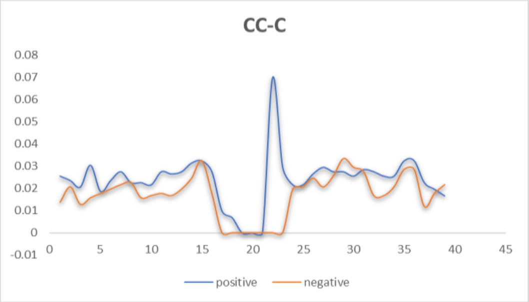
CC-C
0.08
0.07
0.06
0.05
0.04
0.03
0.02
0.01
0
-0.01
0 5 10 15 20 25 30 35 40 45
positive
negative

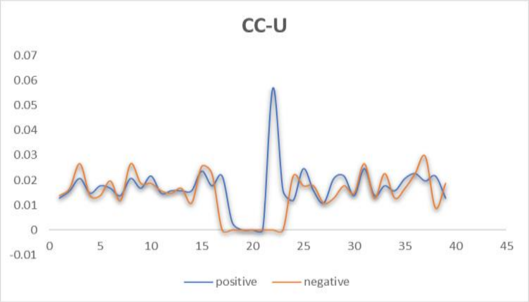
CC-U
0.07
0.06
0.05
0.04
0.03
0.02
0.01
0
-0.01
0 5 10 15 20 25 30 35 40 45
positive
negative

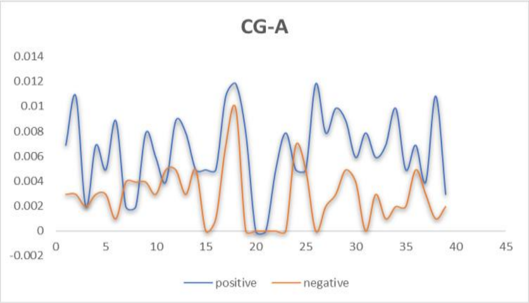
CG-A
0.014
0.012
0.01
0.008
0.006
0.004
0.002
0
-0.002
0 5 10 15 20 25 30 35 40 45
positive negative

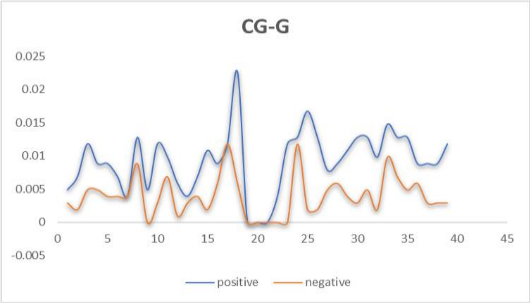
CG-G
0.025
0.02
0.015
0.01
0.005
0
-0.005
0 5 10 15 20 25 30 35 40 45
positive negative

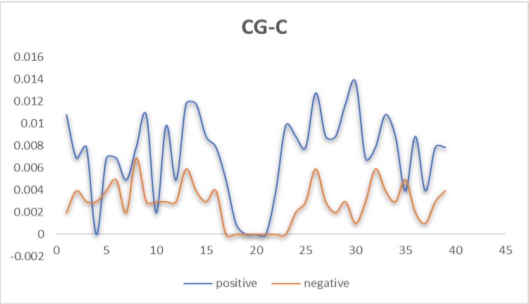
CG-C
0.016
0.014
0.012
0.01
0.008
0.006
0.004
0.002
0
-0.002
0 5 10 15 20 25 30 35 40 45
positive negative

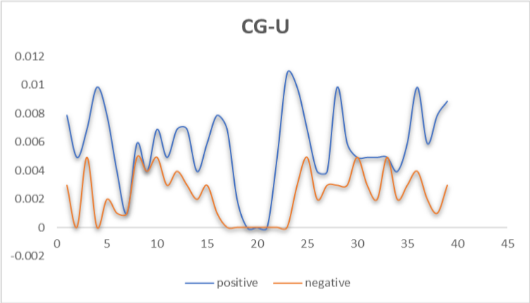
CG-U
0.012
0.01
0.008
0.006
0.004
0.002
0
-0.002
0 5 10 15 20 25 30 35 40 45
positive negative

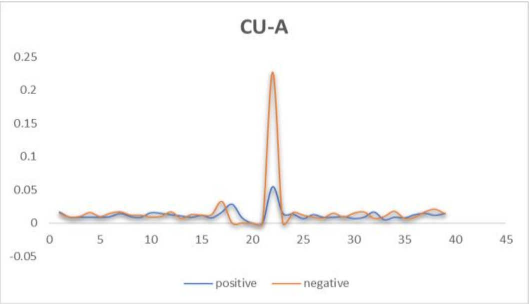
CU-A
0.25
0.2
0.15
0.1
0.05
0
-0.05
0 5 10 15 20 25 30 35 40 45
positive negative

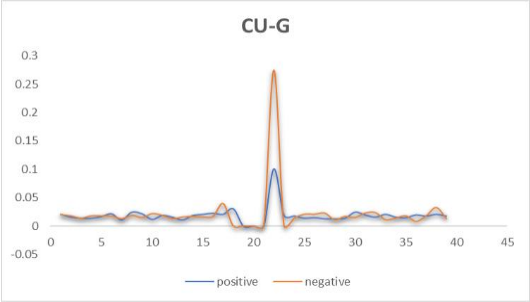
CU-G
0.3
0.25
0.2
0.15
0.1
0.05
0
-0.05
0 5 10 15 20 25 30 35 40 45
positive negative

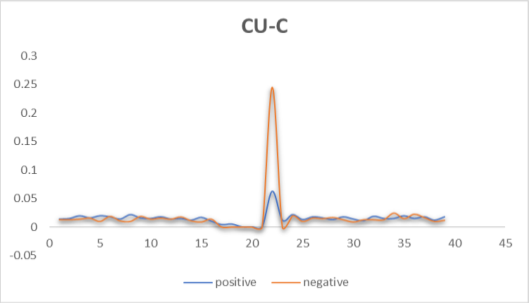
CU-C
0.3
0.25
0.2
0.15
0.1
0.05
0
-0.05
0 5 10 15 20 25 30 35 40 45
positive negative

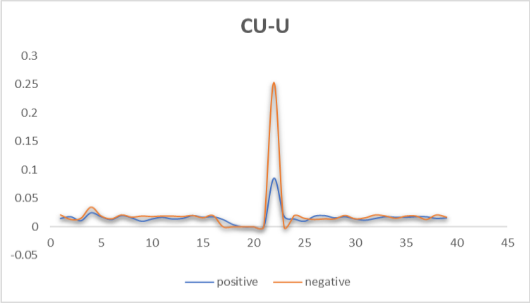
CU-U
0.3
0.25
0.2
0.15
0.1
0.05
0
-0.05
0 5 10 15 20 25 30 35 40 45
positive negative

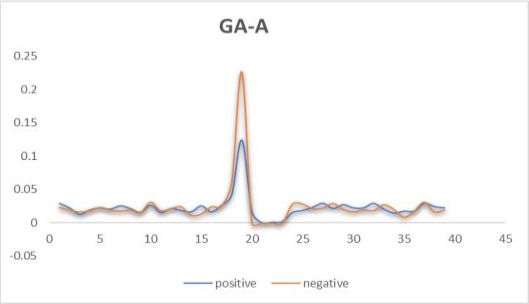
GA-A
0.25
0.2
0.15
0.1
0.05
0
-0.05
0 5 10 15 20 25 30 35 40 45
positive negative

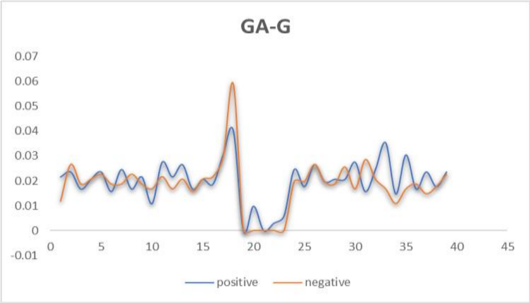
GA-G
0.07
0.06
0.05
0.04
0.03
0.02
0.01
0
-0.01
0 5 10 15 20 25 30 35 40 45
positive negative

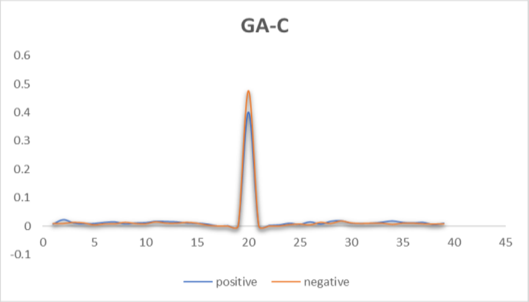
GA-C
0.6
0.5
0.4
0.3
0.2
0.1
0
-0.1
0 5 10 15 20 25 30 35 40 45
positive negative

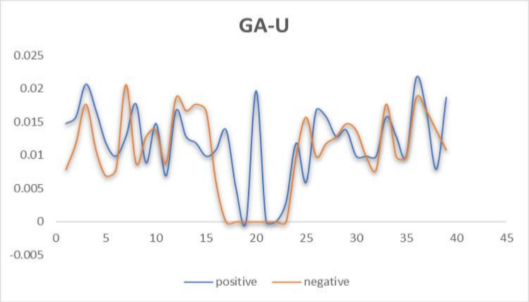
GA-U
0.025
0.02
0.015
0.01
0.005
0
-0.005
0 5 10 15 20 25 30 35 40 45
positive negative

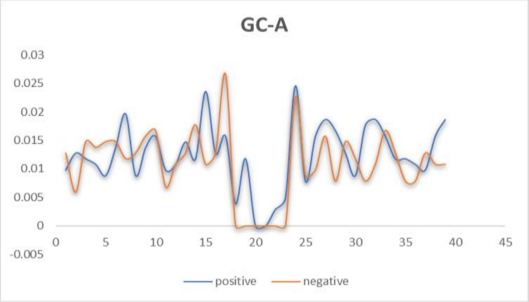
GC-A
0.03
0.025
0.02
0.015
0.01
0.005
0
-0.005
0 5 10 15 20 25 30 35 40 45
positive negative

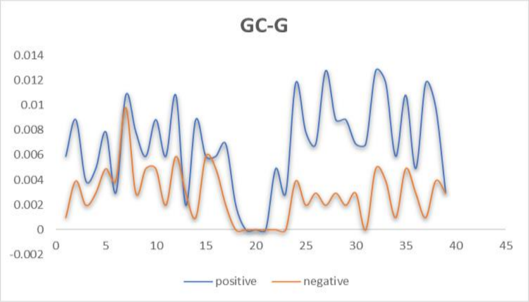
GC-G
0.014
0.012
0.01
0.008
0.006
0.004
0.002
0
-0.002
0 5 10 15 20 25 30 35 40 45
positive negative

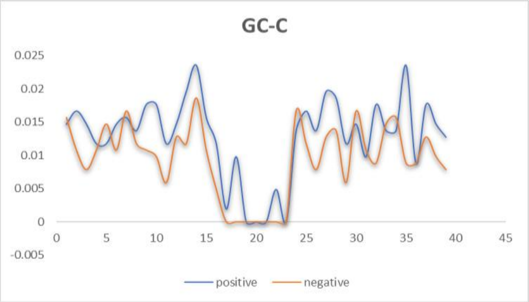
GC-C
0.025
0.02
0.015
0.01
0.005
0
-0.005
0 5 10 15 20 25 30 35 40 45
positive negative

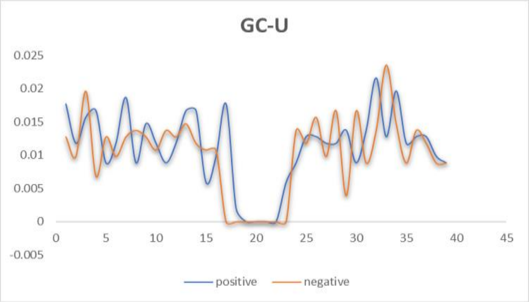
GC-U
0.025
0.02
0.015
0.01
0.005
0
-0.005
0 5 10 15 20 25 30 35 40 45
positive negative

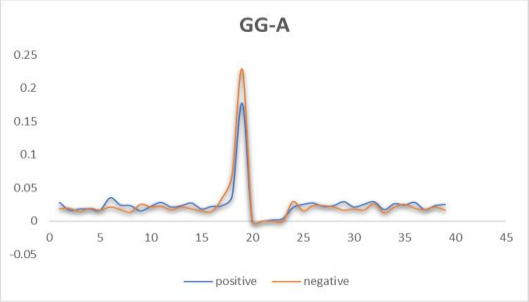
GG-A
0.25
0.2
0.15
0.1
0.05
0
-0.05
0 5 10 15 20 25 30 35 40 45
positive negative

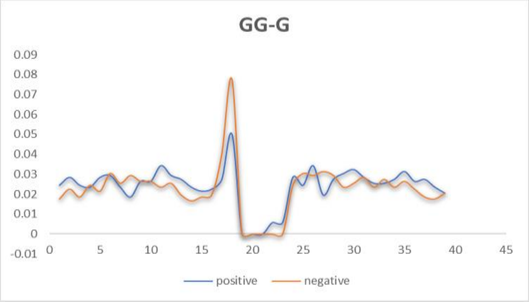
GG-G
0.09
0.08
0.07
0.06
0.05
0.04
0.03
0.02
0.01
0
-0.01
0 5 10 15 20 25 30 35 40 45
positive negative

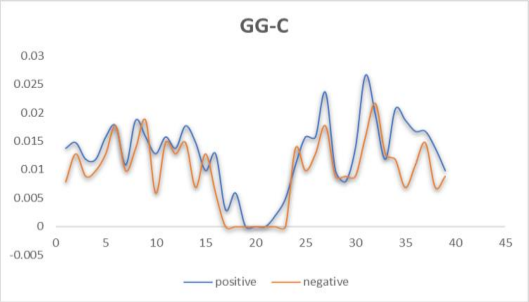
GG-C
0.03
0.025
0.02
0.015
0.01
0.005
0
-0.005
0 5 10 15 20 25 30 35 40 45
positive negative

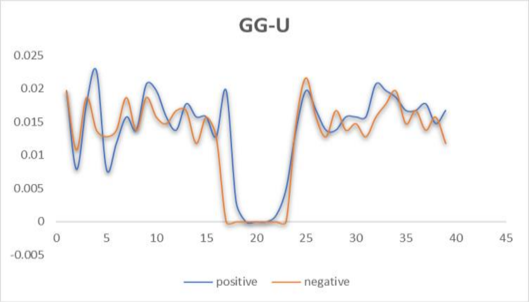
GG-U
0.025
0.02
0.015
0.01
0.005
0
-0.005
0 5 10 15 20 25 30 35 40 45
positive negative

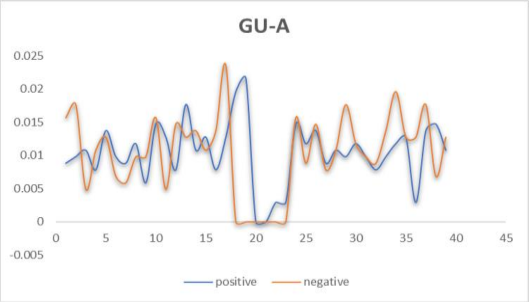
GU-A
0.025
0.02
0.015
0.01
0.005
0
-0.005
0 5 10 15 20 25 30 35 40 45
positive
negative

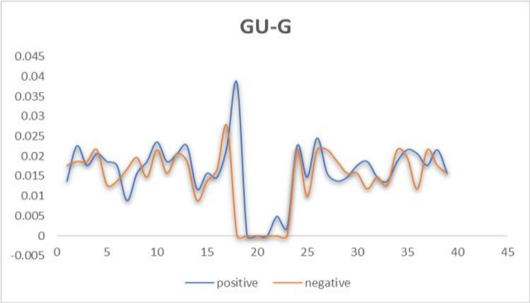
GU-G
0.045
0.04
0.035
0.03
0.025
0.02
0.015
0.01
0.005
0
-0.005
0 5 10 15 20 25 30 35 40 45
positive
negative

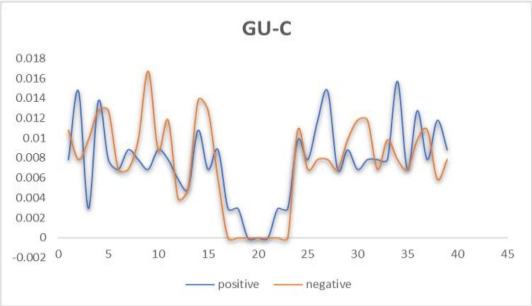
GU-C
0.018
0.016
0.014
0.012
0.01
0.008
0.006
0.004
0.002
0
-0.002
0 5 10 15 20 25 30 35 40 45
positive
negative

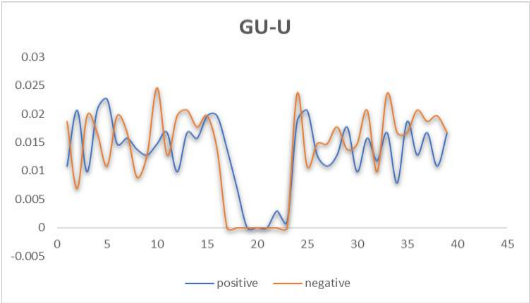
GU-U
0.03
0.025
0.02
0.015
0.01
0.005
0
-0.005
0 5 10 15 20 25 30 35 40 45
positive
negative

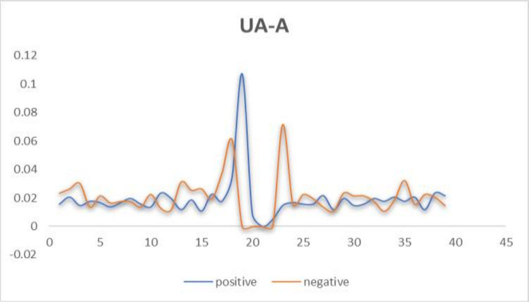
UA-A
0.12
0.1
0.08
0.06
0.04
0.02
0
-0.02
0 5 10 15 20 25 30 35 40 45
positive
negative

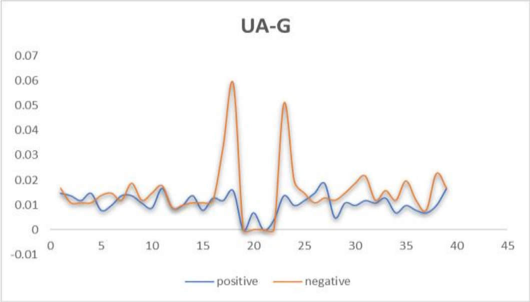
UA-G
0.07
0.06
0.05
0.04
0.03
0.02
0.01
0
-0.01
0 5 10 15 20 25 30 35 40 45
positive
negative

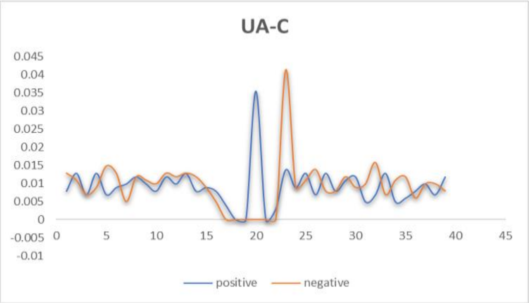
UA-C
0.045
0.04
0.035
0.03
0.025
0.02
0.015
0.01
0.005
0
-0.005
-0.01
0 5 10 15 20 25 30 35 40 45
positive
negative

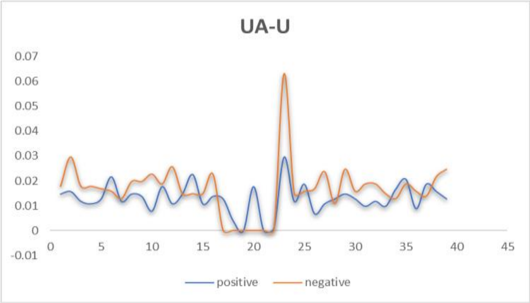
UA-U
0.07
0.06
0.05
0.04
0.03
0.02
0.01
0
-0.01
0 5 10 15 20 25 30 35 40 45
positive
negative

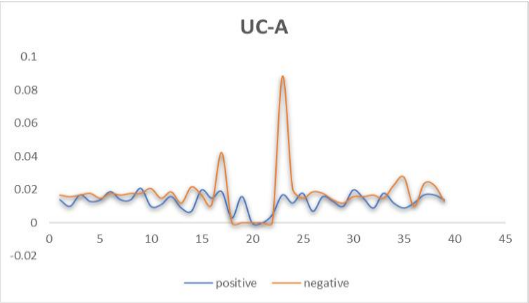
UC-A
0.1
0.08
0.06
0.04
0.02
0
-0.02
0 5 10 15 20 25 30 35 40 45
positive
negative

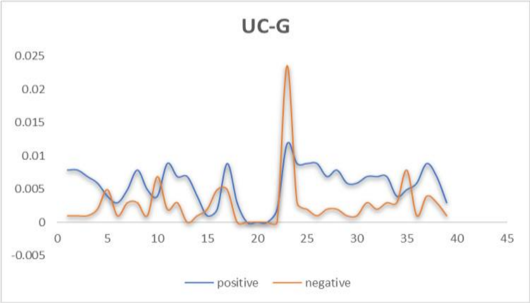
UC-G
0.025
0.02
0.015
0.01
0.005
0
-0.005
0 5 10 15 20 25 30 35 40 45
positive
negative

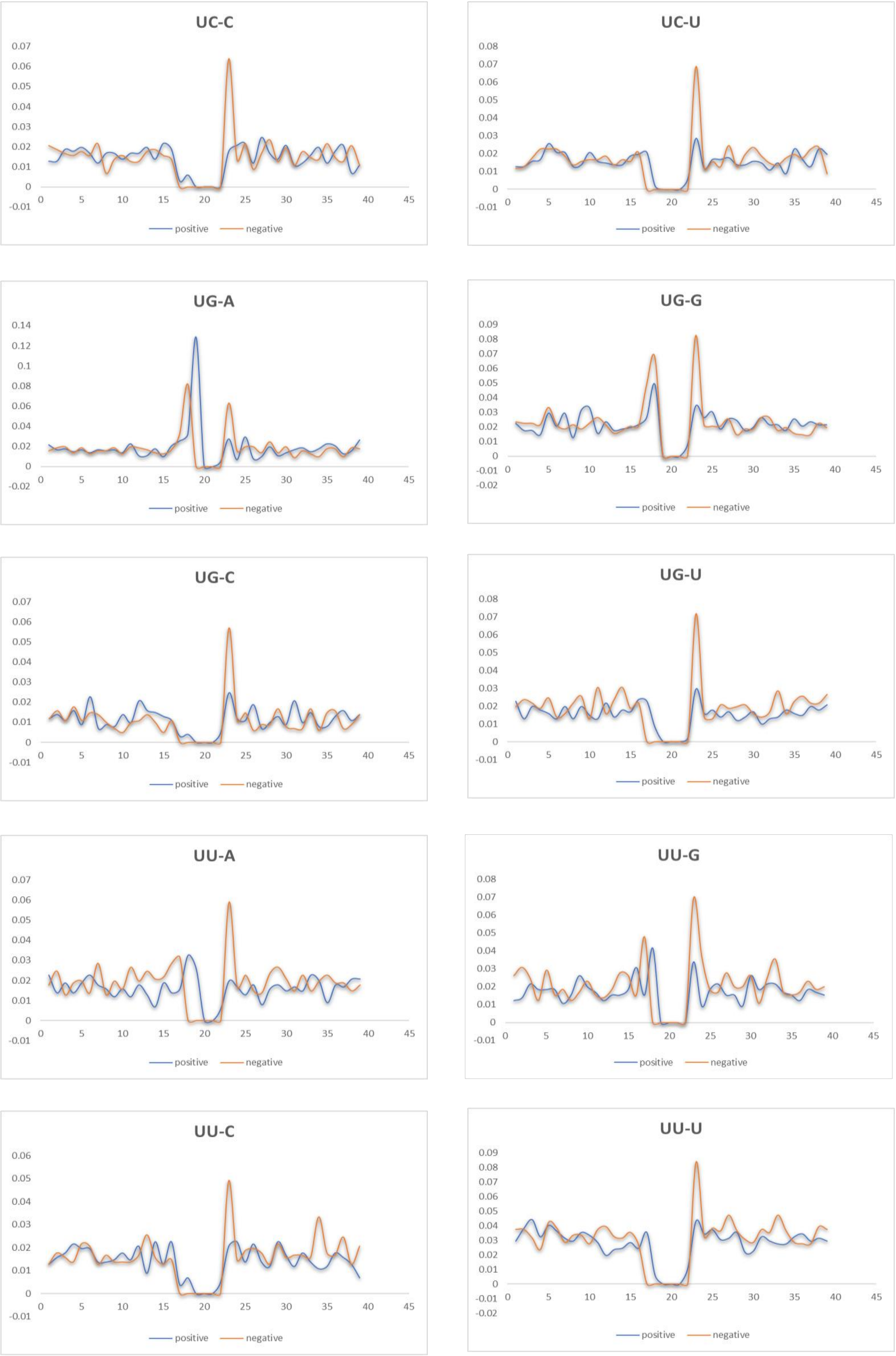

Figure S6 Second-order transition probabilities of Homo sapiens data between adjacent nucleotides at all sites. The blue and red lines represent the m6A and non-m6A sequences, respectively.
